# Prussian Blue Analogues in Aqueous Batteries and Desalination Batteries

**DOI:** 10.1007/s40820-021-00700-9

**Published:** 2021-08-05

**Authors:** Chiwei Xu, Zhengwei Yang, Xikun Zhang, Maoting Xia, Huihui Yan, Jing Li, Haoxiang Yu, Liyuan Zhang, Jie Shu

**Affiliations:** School of Materials Science and Chemical Engineering, Ningbo University, Ningbo, 315211 Zhejiang People’s Republic of China

**Keywords:** Prussian blue analogues, Electrochemistry, Cathode, Aqueous batteries, Desalination batteries

## Abstract

In the applications of large-scale energy storage, aqueous batteries are considered as rivals for organic batteries due to their environmentally friendly and low-cost nature. However, carrier ions always exhibit huge hydrated radius in aqueous electrolyte, which brings difficulty to find suitable host materials that can achieve highly reversible insertion and extraction of cations. Owing to open three-dimensional rigid framework and facile synthesis, Prussian blue analogues (PBAs) receive the most extensive attention among various host candidates in aqueous system. Herein, a comprehensive review on recent progresses of PBAs in aqueous batteries is presented. Based on the application in different aqueous systems, the relationship between electrochemical behaviors (redox potential, capacity, cycling stability and rate performance) and structural characteristics (preparation method, structure type, particle size, morphology, crystallinity, defect, metal atom in high-spin state and chemical composition) is analyzed and summarized thoroughly. It can be concluded that the required type of PBAs is different for various carrier ions. In particular, the desalination batteries worked with the same mechanism as aqueous batteries are also discussed in detail to introduce the application of PBAs in aqueous systems comprehensively. This report can help the readers to understand the relationship between physical/chemical characteristics and electrochemical properties for PBAs and find a way to fabricate high-performance PBAs in aqueous batteries and desalination batteries.

## Highlights

- The characteristics of Prussian blue analogues PBAs in different aqueous systems are analyzed.
- The relationship between structure and performance of PBAs is summarized.
- The measures to improve electrochemical property of PBAs are proposed.

## Graphical abstract

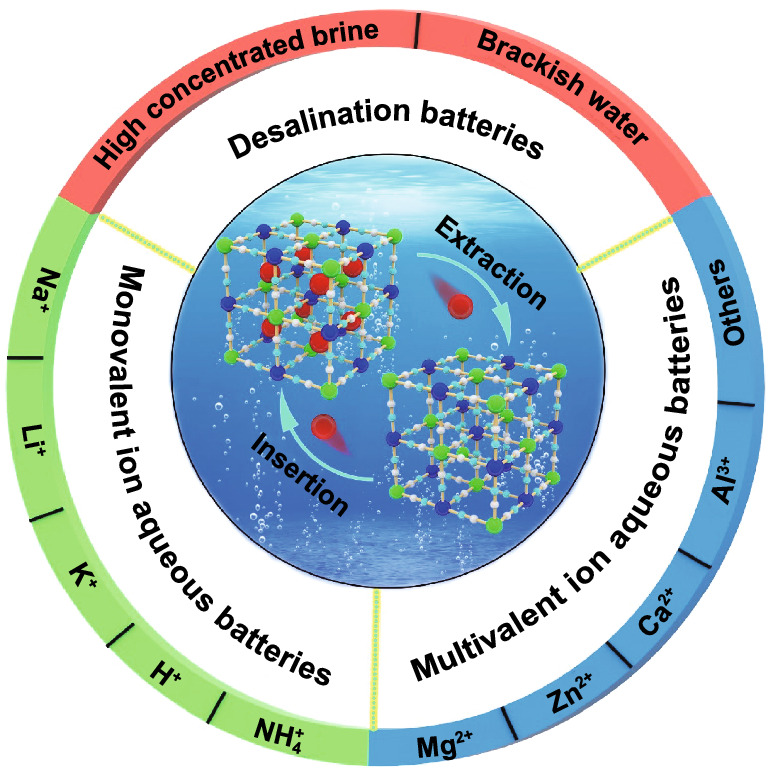

## Introduction

As is known to all, energy is the source of economic development and the material basis for human survival. Therefore, energy has always been a common concern of the world. Traditional energy sources have brought many changes to the development of human society and the world. However, the excessive use of traditional energy produces carbon dioxide and other pollutants, causing serious ecological problems. To solve these environmental problems, scientists are beginning to explore renewable energy sources to reduce environmental pollution and meet the needs of social development [1–5]. However, these clean energy sources generally have the disadvantages of location dependence [6–10], high cost and low efficiency, which restrict their large-scale applications [11–16]. In this case, a lot of attentions have been paid to the developments of rechargeable batteries [17–22], fuel cells [23–29] and supercapacitors [30–35], among which the rechargeable batteries have received the most attention. For rechargeable batteries, most of them are powered by organic electrolytes due to high voltage, high energy density and wide electrochemical window of organic electrolyte systems [36–41]. However, the volatilization, toxicity and flammability of organic electrolytes have brought many environmental and safety problems to their widespread applications [42, 43], especially in large-scale electrical storage applications. To get better batteries, much effort has been paid to aqueous electrolytes due to their advantages of high safety, environmental friendliness and low cost [44–46].

**Fig. 1:**
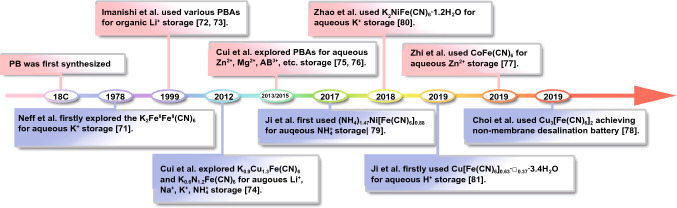
A summary of the key milestone in the development of PBAs used as electrode material in aqueous batteries

Aside from electrolytes, the selection of electrode material also occupies an important position in the application of energy storage devices. In various types of electrode materials, tunnel- and layered-type transition metal oxides [47–53], polyanionic compounds [54–59], PBAs [60–70] have been widely studied. In recent researches, the most commonly used cathode materials are metal oxides because of their high theoretical capacity. However, this material has two disadvantages. One is that the synthesis method is complex, and the other is that many metal oxides are unable to resist the erosion of corrosive electrolytes, which are usually characterized by low rate capacity and poor structural stability. Polyanionic compounds as typical electrode materials possess high safety, low cost and environmental friendliness. However, their poor electrochemical properties restrict the practical application. PBAs are blessed with the advantages of acceptable theoretical capacity, high stability and low cost. In particular, many PBAs can be synthesized by simple methods, and nano-structured materials are usually obtained by the co-precipitation method. Therefore, these materials have great application prospects in rechargeable batteries. In the early eighteenth century, the PB was first synthesized in Berlin, but is initially used as a pigment. With the development of energy storage technology, the requirement of the low cost of the electrode materials promotes the application of PB in the battery industry. In 1978, Neff et al*.* first explored the reversible inserted/extracted electrochemical property of K^+^ in K_2_Fe^II^Fe^II^(CN)_6_ and opened the door of PBAs as electrode material [71]. Then, in 1999, the various types of PBAs used as electrode in organic Li^+^ battery are reported [72, 73]. However, the extensive research of PBAs as electrode material in aqueous batteries began to rise after the twenty-first century. In 2012, Cui et al*.* proved that Li^+^, Na^+^, K^+^ and NH_4_^+^ exhibit reversible inserted/extracted ability in K_0.9_Cu_1.3_Fe(CN)_6_ and K_0.6_Ni_1.2_Fe(CN)_6_, respectively, which inspires the research interest for PBAs in aqueous battery [74]. Aside from these monovalent ions, they later demonstrated that the multivalent ions (Zn^2+^, Mg^2+^, Al^3+^, etc*.*) can also be reversibly inserted/extracted in PBAs [75, 76]. Especially for aqueous multivalent-ion batteries, Zhi et al. recently found that CoFe(CN)_6_ shows the high capacity and long cycle performance in aqueous Zn^2+^ batteries, which further promoted the research of PBA in aqueous multivalent ions batteries [77]. Meanwhile, the application of PBAs in desalination battery as a kind of aqueous battery also has attracted more and more attention in recent years; particularly, it has great development potential in realizing the non-membrane of desalination battery [78]. To understand the development process of PBAs in aqueous batteries more intuitively, we also summarized other milestone applications of PBAs in aqueous batteries in the timeline in Fig. 1.

Obviously, PBAs have been widely studied as battery electrode material for some years [71–81], so there are some relevant reviews on the application of PBAs in the battery field. For example, Li and co-workers introduced the application of PBAs in sodium-ion and potassium-ion batteries in detail, and some strategies for performance optimization are proposed [82]. Srinivasan discussed the application of various electrode materials, including PBAs, in aqueous Zn^2+^ and Al^3+^ batteries [83]. Ji and co-workers introduced the PBAs as electrodes for aqueous monovalent-ion batteries [84]. Zhi and co-workers analyzed the PBAs as host materials for various carriers in both organic and aqueous electrolyte [85]. However, none of these reviews systematically discusses the application of PBAs in aqueous batteries. In this review, we comprehensively introduce the application of PBAs in aqueous monovalent-ion batteries and multivalent-ion batteries. Impressively, the electrochemical properties of various PBAs and their structural characteristics are discussed. Meanwhile, various aqueous desalination batteries with unique characteristics are also introduced because they are working with the same mechanism as aqueous batteries. Therefore, this paper has a high reference value for future research on the PBAs used as electrode materials in aqueous batteries and desalination batteries.

## PBAs in Aqueous Batteries

**Fig. 2:**
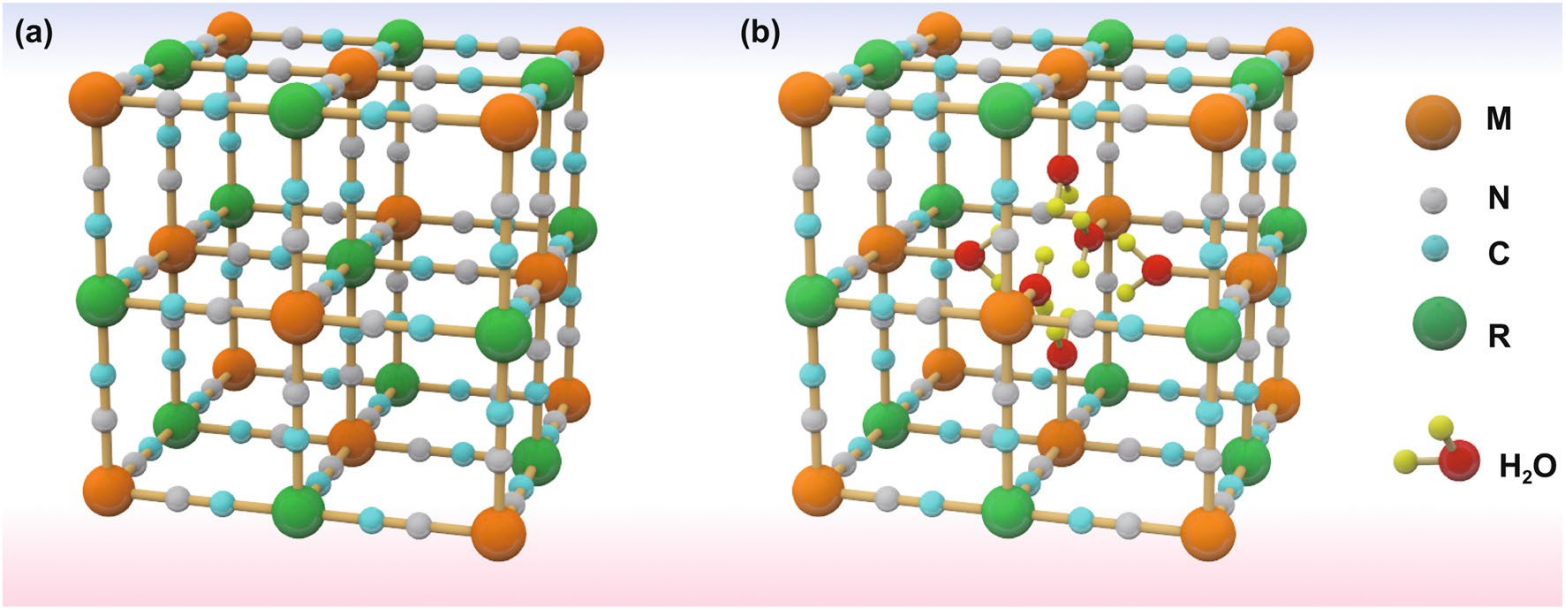
**a** Schematic crystal structure of PBAs frameworks: An intact A_x_M[R(CN)_6_] framework without structural defects. **b** A defective A_x_M[R(CN)_6_] framework with vacancies and interstitial water existing in each unit cell

With a metal–organic open framework, PBAs possess a general composition of A_x_M[R(CN)_6_]·wH_2_O in which A is the alkali metals and M and R represent the transition metal. It is worth noting that most of the R-site in PBAs for the aqueous battery is replaced by Fe atom. Here, the classic PBAs structure is illustrated in Fig. 2. In this face-centered cubic structure, the nitrogen and carbon atoms from the CN ligands combine with the M and R atom, respectively, creating open ionic channels and spacious interstitial spaces [86]. As electrode material used in aqueous batteries, PBAs release and store energy through the insertion and extraction of host ions in PBAs. Here, A_x_M[R(CN)_6_]·wH_2_O is taken for example as a cathode. During the discharging process, host ions in the electrolyte are inserted into PBAs, which causes the R or M and R atoms to simultaneously undergo a reduction reaction to release energy. In the charging process, host ions are extracted from PBAs with R or M and R atoms simultaneous undergoing oxidation reaction to store energy. The charging and discharging process can be reflected by the following chemical reaction equation:

1 $${\text{A}}_{x} {\text{M}}\left[ {{\text{R}}\left( {{\text{CN}}} \right)_{{6}} } \right] \cdot {\text{wH}}_{{2}} {\text{O}} \leftrightarrow {\text{xA}}^{ + } {\text{ + M[R(CN)}}_{{6}} {]}^{ - x} \cdot {\text{wH}}_{{2}} {\text{O}}{.}$$

**Fig. 3:**
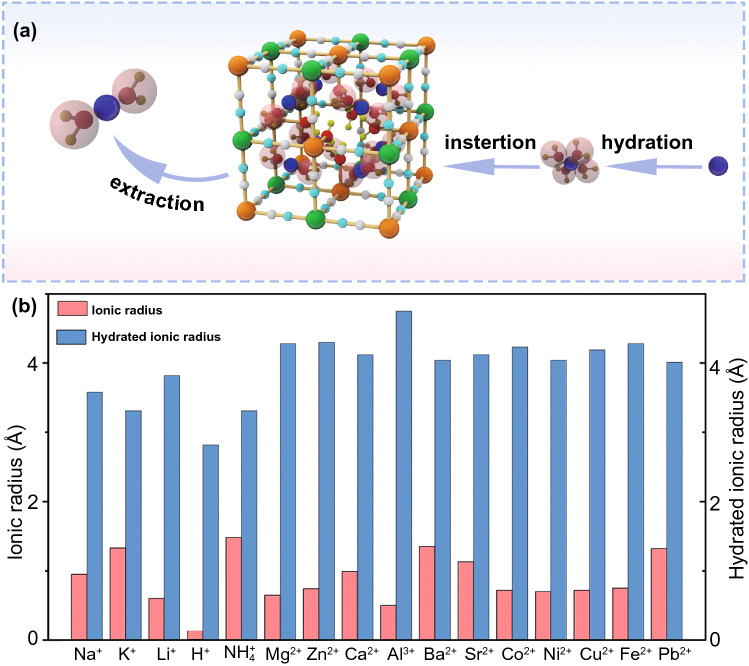
**a** Schematic of host ion insertion and extraction in PBAs. **b** Histogram of ionic radius and hydration radius of various host ions

Furthermore, Fig. 3a also shows the insertion and extraction process of host ions in PBAs. For the aqueous battery, host ions will undergo a hydration process before entering into PBAs and then dehydrate after entering into PBAs. As shown in Fig. 3b, the hydration ion radius will be larger than the original ion radius after hydration, which puts forward higher requirements for the structural stability of PBAs.

**Fig. 4:**
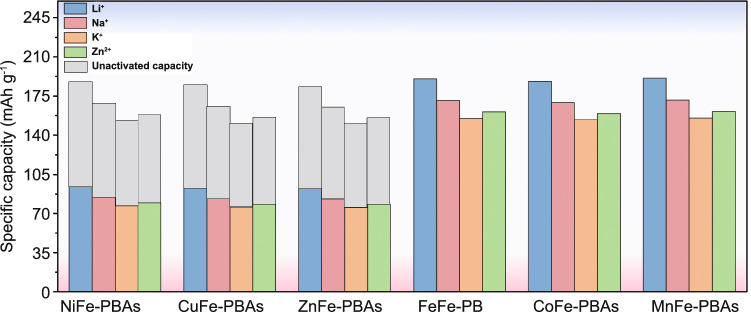
Theoretical capacity of different PBAs in various aqueous batteries (the shaded part represents the un-activated capacity of the single-atom redox PBAs)

**Fig. 5:**
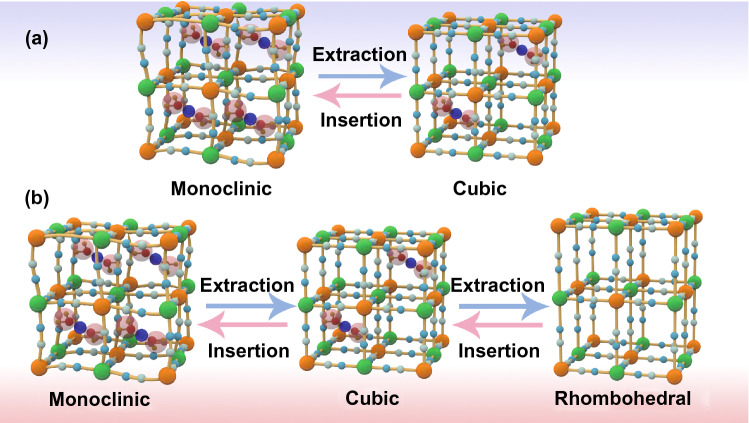
Structure transformation of PBAs **a** between monoclinic and cubic structures and **b** among monoclinic, cubic and rhombohedral structures

For PBAs, it possesses the 3D cubic structure and tunable chemical compositions that give PBAs some advantages when inserting ions with large hydrated ionic radius. Firstly, there are many available “A” sites with large interstitial (~ 4.6 Å diameter) in the body center of PBAs lattice. Meanwhile, the PBAs possess a special ions channel with approximately 3.2 Å diameter in the (100) direction [87, 88]. The large insertion site and ion channel ensure the fast reaction kinetics of most host ions with small hydrated radius when they are inserted/extracted in/from PBAs, which usually brings well cycling stability to PBAs [89, 90]. Secondly, PBAs exhibit multivalent redox-active centers such as the M^3+^/M^2+^ and Fe^3+^/Fe^2+^ couples, which means that PBAs can provide more capacity. The theoretical capacities of different PBAs in characteristic aqueous systems are summarized in Fig. 4. For FeFe-PB, CoFe-PBAs and MnFe-PBAs, their theoretical capacities are all over 150 mAh g^−1^. But the theoretical capacity utilization of NiFe-PBAs, CuFe-PBAs and ZnFe-PBAs is only 50% because Ni, Cu and Zn in PBAs are electrochemically inert. In addition to these structural advantages, PBAs also have some structural defects that need to be addressed. During the preparation process of PBAs through co-precipitation, it will inevitably bring some interstitial water and vacancies in PBAs crystals, and Fig. 2 exhibits the crystal structure diagram of PBAs with vacancies and interstitial water. For interstitial water, it will cause the following two disadvantages to PBAs. First of all, the presence of interstitial water will occupy the insertion site of host ions, resulting in a capacity decrease of PBAs [91, 92]. Secondly, interstitial water in PBAs may enter into the electrolyte in the process of ion insertion and extraction, resulting in the decrease of electrolyte concentration that will aggravate the dissolution of PBAs structure. For Fe(CN)_6_ vacancies, its random distribution can cause the distortion of PBAs lattice, thus reducing the structural stability. Especially for reduced-form PBAs, due to the oxidation reaction of transition metal elements, the structural instability is more serious. Furthermore, as shown in Fig. 5a, in the process of host ions insertion/extraction in PBAs, its structure will change from cubic structure to monoclinic structure, which reduces the stability of PBAs. Generally, it is concluded that the host ions-deficient PBAs present the cubic phase, while host ions-rich PBAs usually present the monoclinic phase. Impressively, the asymmetrical occupation of electrons from transition metal atom in the degenerate orbitals will produce John–Teller effect in PBAs structure. This phenomenon will cause different shielding effects of electrons in different directions on central ions, thus distorting the geometric configuration of PBAs molecules, reducing the symmetry and degeneracy of the orbitals and finally reducing the energy of the system. Figure 5b exhibits the phase transitions between monoclinic, cubic and rhombohedral structures caused by John–Teller distortion. Despite the presence of these vacancies and interstitial water in PBAs, a large number of studies have shown that their content can be well controlled by a controlled crystallization method. Besides, the John–Teller distortion can also be inhibited by partial substitution of the transition metal attached to N by other metal ion. Thus, PBAs are suitable electrode materials for aqueous batteries. Herein, for the sake of better introducing the application of PBAs in aqueous batteries, we review the recent advances of PBAs in aqueous monovalent-ion batteries and multivalent-ion batteries, respectively.

### PBAs for Aqueous Monovalent-Ion Batteries

The application of PBAs in aqueous monovalent-ion batteries can be divided into different fields: aqueous Na^+^, Li^+^, K^+^, H^+^ and NH_4_^+^ batteries. For aqueous Na^+^ and K^+^ batteries, PBAs usually show excellent cyclic stability due to the better structural compatibility and moderate hydrated radius for Na^+^ and K^+^. Usually, these PBAs in aqueous Na^+^ and K^+^ batteries can maintain more than 80% of the initial capacity within 300 cycles. Although PBAs have poor cyclic stability in aqueous Li^+^ batteries due to the large hydration ion radius of Li^+^ (higher than Na^+^, K^+^, H^+^, and NH_4_^+^), mixed-ion batteries composed of Li^+^ and other metal ions have great application prospects in the improvement in energy density. Besides, the excellent rate performance and cycling stability of PBAs in aqueous H^+^ batteries and aqueous NH_4_^+^ batteries have also attracted more and more attention in recent years due to the special transport mechanism of H^+^ in aqueous electrolyte and the good structural compatibility of NH_4_^+^ and PBAs. This section will comprehensively introduce the application of PBAs in these aqueous monovalent-ion batteries.

#### ***PBAs for Aqueous Na***^+^***Batteries***

For the PBAs in aqueous Na^+^ batteries (ASIB), they can be divided into single-metal-atom redox PBAs (NiFe-PBAs, CuFe-PBAs and ZnFe-PBAs) and double-metal-atom redox PBAs (FeFe-PB, CoFe-PBAs and MnFe-PBAs). The single-metal-atom redox PBAs mean that only Fe atom can undergo the redox reaction and provide capacity during the insertion and extraction of Na^+^. Therefore, as shown in Fig. 4, the theoretical capacity utilization of the single-metal-atom redox PBAs is only 50%. The double-metal-atom redox PBAs means that both Fe atom and M atom can undergo redox reaction and provide capacity during the insertion and extraction of Na^+^. Herein, for better comparison, PBAs in ASIB are cataloged into two groups: PBAs with single-atom redox and PBAs with double-atom redox.

##### PBAs with Single-Atom Redox

From the above introduction, when the Ni, Cu, and Zn are located at M-site in A_x_M[Fe(CN)_6_]_y_·nH_2_O, these types of PBAs belong to the single-metal-atom redox PBAs. The typical single-metal-atom redox nickel hexacyanoferrate (K_0.6_Ni_1.2_Fe(CN)_6_·3.6H_2_O, NiFe-PBA) was firstly synthesized by Cui and co-workers through spontaneous precipitation in aqueous solution as electrode materials for ASIB [43]. As only Fe^3+^/Fe^2+^ redox couple possess electrochemical activity, its discharge capacity only has 60 mAh g^−1^ at the current rate of approximately 0.8 C. However, K_0.6_Ni_1.2_Fe(CN)_6_·3.6H_2_O exhibits excellent electrochemical stability with almost no capacity fading at 8.3 C after 5000 cycles due to the small structural changes and structural stress–strain in the Na^+^ insertion/extraction process. But it is in a Na-deficient state, making it impossible to construct a practical ASIB with conventional Na-free anodes [93]. Generally speaking, a Na insertion cathode should be designed in a Na-rich state (a discharged state), so as to act as a Na^+^ reservoir to provide removable Na^+^ for the Na^+^-deficient anode, thus enabling a rocking-chair Na-ion battery. To address this problem, a Na-rich Na_1.94_Ni_1.03_Fe(CN)_6_·4.8H_2_O was synthesized by Yang and co-workers and construct a practical ASIB with NaTi_2_(PO_4_)_3_ [94].

**Fig. 6:**
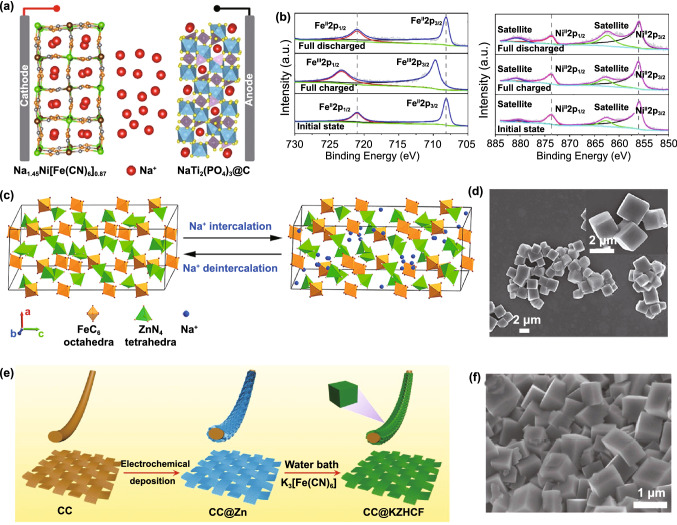
**a** Schematic illustration of Na_1.45_Ni[Fe(CN)_6_]_0.87_·3.02H_2_O/NaTi_2_(PO_4_)_3_ full cell. **b** Ex situ XPS of Fe 2p (left) and Ni 2p (right) spectra. Reproduced with permission from Ref. [95]. Copyright 2020, Elsevier. **c** Schematic illustration of sodium-ion intercalation mechanism in Zn_3_[Fe(CN)_6_]_2_. **d** SEM images of the pristine Zn_3_[Fe(CN)_6_]_2_. Reproduced with permission from Ref. [100]. Copyright 2018, Elsevier. **e** Schematic fabrication process of K_2_Zn_3_[Fe(CN)_6_]_2_·9H_2_O on carbon cloth. **f** SEM image of K_2_Zn_3_[Fe(CN)_6_]_2_·9H_2_O. Reproduced with permission from Ref. [101]. Copyright 2019, Wiley–VCH

Later, Zhu and co-workers also synthesized Na-rich Na_1.45_Ni[Fe(CN)_6_]_0.87_·3.02H_2_O and construct a practical ASIB with NaTi_2_(PO_4_)_3_ (Fig. 6a). Besides, they find monoclinic Na_1.45_Ni[Fe(CN)_6_]_0.87_·3.02H_2_O has advantages in reversible capacity and cycle stability compared with cubic Na_1.21_Ni[Fe(CN)_6_]_0.86_·3.21H_2_O [95]. The reasons can be concluded into three points. Firstly, the monoclinic crystal structure possesses more sodium that makes it contain fewer crystal defects in the lattice. Secondly, during the synthesis of monoclinic Na_1.45_Ni[Fe(CN)_6_]_0.87_·3.02H_2_O, the addition of chelating agents and surfactants reduces the crystallization rate and makes the crystal shape of monoclinic Na_1.45_Ni[Fe(CN)_6_]_0.87_·3.02H_2_O more uniform, thus increasing the contact area between the electrode and the electrolyte and shortening the migration path of ions. Thirdly, a slower reaction rate leads to the formation of higher crystallinity product with less interstitial water and vacancy, thus accelerating Na^+^ insertion/extraction rate. Moreover, an ex situ X-ray photoelectron spectroscope (XPS) at different charge/discharge states in Fig. 6b explored the valance state change of Fe and Ni atoms during Na^+^ extraction/insertion. The result shows that the valance change only occurs in Fe atom, which fully explained the Ni atom in NiFe-PBAs with no electrochemical activity. Although NiFe-PBAs show acceptable performance in terms of electrochemical stability, its low redox potential is not suitable for aqueous energy storage applications.

For the redox potential (E) of host ions in PBAs, there is a qualitative relationship [96]:

2 $$E = E_{{{\text{redox}}}} + E_{{\text{ion - host}}} + E_{{\text{ion - ion}}} .$$

The value of *E*_redox_ is related to the potential of the redox couple itself. *E*_ion-host_ is related to the interaction between host ions and PBAs framework, which usually increases as the radius of the ion enlarges. *E*_ion-ion_ is related to the repulsion between host ions in PBAs. Since the redox couple of single-metal-atom redox PBAs is only [Fe(CN)_6_]^3−^/[Fe(CN)_6_]^4−^, the redox potential will not differ much between them when the same ion is inserted in PBAs. However, the increase of electrons in the antibonding orbital (*e*_g_) of M atom in PBAs can cause [Fe(CN)_6_]^3−^/[Fe(CN)_6_]^4−^ redox potential to move toward more positive values [97]. Thus, the redox potential *vs.* standard hydrogen electrode (SHE, all the potentials in this manuscript are reported versus the SHE) of these single-metal-atom redox PBAs follows the order: ZnFe-PBA > CuFe-PBA > NiFe-PBA. Obviously, CuFe-PBA and ZnFe-PBA are more suitable as cathode material compared with NiFe-PBA in terms of redox potential. The typical Na-rich Na_2_CuFe(CN)_6_ with the insertion potential of 0.82 V was synthesized by Wu and co-workers, and a practical ASIB was constructed with NaTi_2_(PO_4_)_3_ [98]. Impressively, the Na_2_CuFe(CN)_6_-NaTi_2_(PO_4_)_3_ full cell shows high operating voltage (1.4 V) due to the high redox potential of Na_2_CuFe(CN)_6_. Combining the high stability of NiFe-PBA with high redox potential of CuFe-PBA, Zhang and co-workers synthesized Ni-substituted copper hexacyanoferrates (Na_2_Cu_1-x_Ni_x_[Fe(CN)_6_]) as cathode for ASIB [99]. For this Na_2_Cu_1-x_Ni_x_[Fe(CN)_6_] cathode, it can exhibit a discharge capacity of 56 mAh g^−1^ with a capacity retention of 96% after 1000 cycles. Impressively, its redox potential can be adjusted from 0.6 to 1.0 V with the increased content of Cu.

For ZnFe-PBAs, it has great potential in improving energy density due to its high redox potential. Recently, Liu and co-workers reported a full cell that uses Zn_3_[Fe(CN)_6_]_2_ as cathode, NaTi_2_(PO_4_)_3_ as anode, and NaClO_4_–H_2_O–polyethylene glycol as electrolyte [100]. Figure 6c, d shows the schematic illustration of Na^+^ intercalation mechanism in Zn_3_[Fe(CN)_6_]_2_ and its basic morphology. For this battery, its energy density can reach up to 59 Wh kg^−1^ due to the high output voltage of 1.6 V. Besides, the energy density of ZnFe-PBAs can be further improved by improving the synthesis method. Yao and co-workers adopt a two-step method of electrodeposition and water bath treatment grew K_2_Zn_3_[Fe(CN)_6_]_2_·9H_2_O on carbon cloth (CC@ZnFe-PBAs) [101]. The schematic fabrication process is exhibited in Fig. 6e, and the as-formed K_2_Zn_3_[Fe(CN)_6_]_2_·9H_2_O cubes are uniformly deposited on carbon cloth (Fig. 6f). Since the CC@ZnFe-PBA electrode avoids the use of inactive substances (conductive additive, binder), it exhibits a high energy density of 14.3 mW cm^−2^ and high output voltage of 1.6 V when it is combined with NaTi_2_(PO_4_)_3_ to form a full cell. In general, the single-metal-atom redox PBAs inherit the advantage of high electrochemical stability that makes it possible to be used as ASIB electrode. However, considering the specific capacity, double-mental-atom redox PBAs are more competitive.

##### PBAs with Double-Atom Redox

Obviously, single-metal-atom redox PBAs have a disadvantage that their capacity is not high. The essential cause of this problem is that only one redox-active couple (Fe^3+^/Fe^2+^) releases its redox capacity. In contrast, double-metal-atom redox PBAs have the advantage in capacity because both Fe^3+^/Fe^2+^ and M^3+^/M^2+^ can provide capacity. As typical double-metal-atom redox PBAs, NaFeFe(CN)_6_ [102] and Co_3_[Fe(CN)_6_]_2_ [103] are initially proposed as host materials for Na^+^ storage. However, its specific capacity is approaching 70 mAh g^−1^ which is much less than the theoretical capacity (Fig. 4). Through reviewing the recent advances of PBAs in ASIB, there are four main reasons for the low capacity of double-metal-atom redox PBAs. Firstly, the disadvantage of the synthesis method results in a lot of Fe(CN) vacancies. Secondly, more interstitial water in PBAs hinders Na^+^ insertion. Thirdly, the low concentration of electrolyte results in a narrow voltage window, which affects the charge/discharge capacity. Finally, M atoms connecting with N atoms have weaker electrochemical activity. To a large extent, the above four factors limit the released capacity of double-metal-atom redox PBAs.

**Fig. 7:**
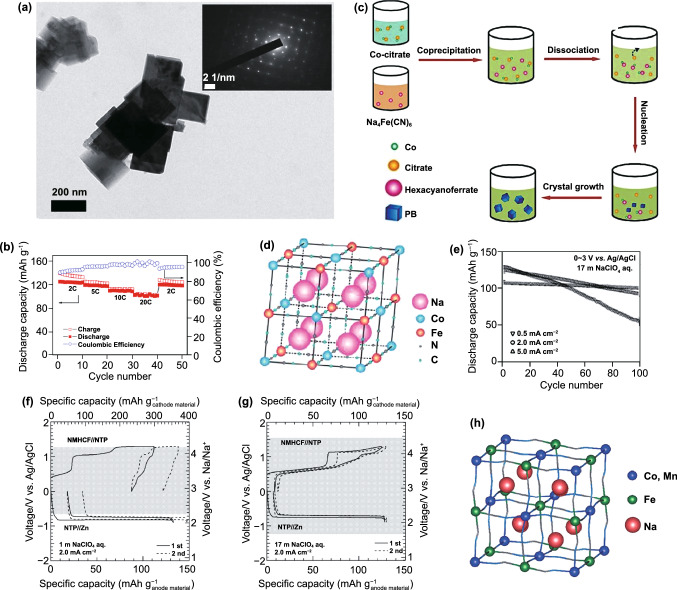
**a** TEM images of as-prepared Na_1.33_Fe[Fe(CN)_6_]_0.82_ nanocrystals. **b** Rate performance of Na_1.33_Fe[Fe(CN)_6_]_0.82_. Reproduced with permission from Ref. [104]. Copyright 2015, Elsevier. **c** Schematic representation of the formation mechanism of Na_2_CoFe(CN)_6_ in a controlled crystallization reaction. **d** Crystal structure of Na_2_CoFe(CN)_6_. Reproduced with permission from Ref. [105]. Copyright 2015, Wiley–VCH. **e** Cyclability dependence on the current density of Na_2_MnFe(CN)_6_ half cell with 17 M NaClO_4_ aqueous electrolyte. The first and second charge/discharge curves of Na_0.13_Mn[Fe(CN)_6_]_0.81_/NaTi_2_(PO_4_)_3_ full cell and NaTi_2_(PO_4_)_3_//Zn half cell **f** in 1 M NaClO_4_ electrolyte and **g** in 17 M NaClO_4_ electrolyte. Reproduced with permission from Ref. [106]. Copyright 2017, Electrochemical Society. **h** Crystal structure of manganese–cobalt hexacyanoferrate with rhombohedral lattice. Reproduced with permission from Ref. [107]. Copyright 2016, The Royal Society of Chemistry

For the control of defects and the amount of interstitial water, Yang and co-workers synthesized Na_1.33_Fe[Fe(CN)_6_]_0.82_ with low defect and water content by a multi-step crystallization method [104]. The cubic morphology is displayed in Fig. 7a. In comparison with hydrated FeFe-PBA, Na_1.33_Fe[Fe(CN)_6_]_0.82_ exhibits a higher specific capacity of 125 mAh g^−1^ at 2 C. Even at 20 C, it still can present a highly efficient Na storage reversibility with a desirable capacity of 102 mAh g^−1^ (Fig. 7b). Later, Yang adopted a similar crystallization method as shown in Fig. 7c to achieve vacancy-free Na_2_CoFe(CN)_6_ (Na_1.85_Co[Fe(CN)_6_]_0.99_·2.5H_2_O) [105], which exhibits perfect crystal structure without vacancy in Fig. 7d. Because of the vacancy-free structure and two redox centers, the capacity of Na_2_CoFe(CN)_6_ can approach up to 130 mAh g^−1^, which is a high value for aqueous battery in existing reports.

Besides the defects and interstitial water of PBAs, electrolyte concentration is also one of the factors affecting the electrochemical performance of PBAs. Take MnFe-PBAs for example, Okada and co-workers synthesized Na_0.13_Mn[Fe(CN)_6_]_0.81_ and explored the influence of electrolyte concentration on electrochemical performance [106]. For Na_0.13_Mn[Fe(CN)_6_]_0.81_/NaTi_2_(PO_4_)_3_ full battery in 1 M NaClO_4_ and 17 M NaClO_4_, they find the electrochemical window in 17 M NaClO_4_ is 2.8 V which is higher than the 1.9 V in 1 M NaClO_4_ and the initial charge/discharge capacities can raise to 124/116 mAh g^−1^ (Fig. 7e). The reason is that the free water molecules in high-concentration electrolyte are less than dilute concentration electrolyte. Thus, the evolution of oxygen and hydrogen is inhibited largely, and the electrochemical window is enlarged. Besides, low-concentration electrolyte also has a certain influence on the structural stability of double-metal-atom redox PBAs. In dilute electrolytes, the oxygen evolution is more severe, which can increase the amount of OH^−^ in the electrolyte. After that, M^n+^ dissolved in the electrolyte reacts with OH^−^ to form precipitation and this irreversible reaction will aggravate the structural collapse of double-metal-atom redox PBAs. For Na_0.13_Mn[Fe(CN)_6_]_0.81_, the structural collapse can be described in the following chemical reaction:

3 $${\text{Na}}_{{{0}{\text{.13}}}} {\text{Mn[Fe}}\left( {{\text{CN}}} \right)_{{6}} {]}_{{{0}{\text{.81}}}} {\text{ + 2NaOH }} \to { 0}{\text{.81Na}}_{{3}} {\text{Mn}}\left[ {{\text{Fe}}\left( {{\text{CN}}} \right)_{{6}} } \right]{\text{ + MnO + H}}_{{2}} {\text{O}}{.}$$

In addition, according to the crystal field theory, the d orbital is subjected to the electrostatic repulsion of the octahedron crystal field and split into t_2g_ and e_2g_ orbitals. Compared with t_2g_ orbitals, the variation of electrons in the e_2g_ orbital has more influence on the crystal structure due to the orientation of the e_2g_ orbitals along the axis of the bond [97]. Because their crystal field fission energy is weaker, these transition metals attached to the N atom tend to gain and lose electrons in the e_2g_ orbital when they undergo redox reactions. In contrast, the transition metal connected to C atom has strong crystal field fission energy, and the gain and loss of electrons occur in the t_2g_ orbital. Therefore, the redox reaction of the transition metal connected with N atom has a great influence on the crystal structure, causing it to contribute less capacity. About the relationship of transition metal atoms connecting with N atoms and electrochemical property of double-mental-atom redox PBAs, Cui et al*.* conducted a systematic study through Na_1.54_Co[Fe(CN)_6_]_0.86_∙γ_0.14_∙2.16H_2_O and Na_1.33_Mn[Fe(CN)_6_]_0.79_∙γ_0.21_∙1.88H_2_O [107]. Figure 7f exhibits the crystal structure of the above two PBAs. They prove that the transition metal connected with C atom plays an important role in the stability of the crystal structure and provides more capacities. On the contrary, the transition metal connected with N atom has worse stability in crystal structure due to the weak N-coordinated crystal field and thus provides fewer capacities. Obviously, it provides a new idea for future research on the mechanism of capacity decline of double-metal-atom redox PBAs.

#### ***PBAs for Aqueous Li***^+^***Batteries***

**Fig. 8:**
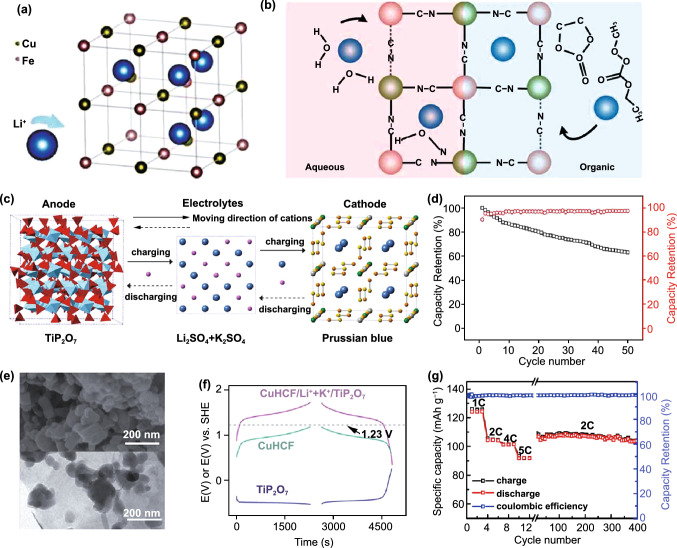
**a** Crystal structure of copper hexacyanoferrate. **b** Schematic of Li^+^ intercalation in copper hexacyanoferrate. Reproduced with permission from Ref. [88]. Copyright 2019, Electrochemical Society. **c** A schematic of K_0.08_Ni_0.75_Zn_0.70_Fe(CN)_6_/TiP_2_O_7_ battery. **d** Cycle life of K_0.08_Ni_0.75_Zn_0.70_Fe(CN)_6_/TiP_2_O_7_ battery at 1 C. Reproduced with permission from Ref. [108]. Copyright 2014, Wiley–VCH. **e** SEM and TEM images of K_0.04_Cu_1.47_Fe(CN)_6_. **f** Galvanostatic profiles of K_0.04_Cu_1.47_Fe(CN)_6_/TiP_2_O_7_ with co-insertion of Li^+^ and K^+^. Reproduced with permission from Ref. [109]. Copyright 2017, The Royal Society of Chemistry. **g** Rate capability and cycling stability of Fe_4_[Fe(CN)_6_]_3_/TiP_2_O_7_ full cell. Reproduced with permission from Ref. [110]. Copyright 2013, American Chemical Society

In aqueous battery system, the host ions inserted in the host material are accompanied by a hydration process in which the free water molecules in the electrolyte will combine with the host ion to form a hydrated ion. After hydration process, the hydrated ionic radius of Li^+^ will get larger. Thus, the insertion performance of Li^+^ in PBAs is usually poor because the large hydrated ionic radius of Li^+^ has a severe impact on the structural stability of PBAs. Earlier research in the insertion of Li^+^ in PBAs also confirmed the theory. In 2012, Cui and co-workers first reported two types of PBAs, K_0.9_Cu_1.3_Fe(CN)_6_ and K_0.6_Ni_1.2_Fe(CN)_6_, and studied their ability to store Li^+^ in aqueous electrolyte [74]. Both PBAs can host Li^+^, but both exhibit poor cycling performance. Later, Lee and co-workers reduced the particle size of copper hexacyanoferrate to nanoscale for achieving Li storage [88]. But the result is still disappointing. Figure 8a, b exhibits the insertion sites and mechanism of Li^+^ in the copper hexacyanoferrate, respectively. It is apparent that the cause of poor Li^+^ insertion ability in PBAs is that in aqueous electrolyte the radius of hydrated Li^+^ is too large. Thus, it is difficult for PBAs to receive hydrated Li^+^ intercalation in the structure and maintain structural stability during the process of Li^+^ insertion and extraction.

Although the Li^+^ storage performance of PBAs in aqueous electrolyte is poor, the co-insertion of Li^+^ with K^+^ or Na^+^ in PBAs has received special attention. In 2014, Liu and co-workers used K_0.08_Ni_0.75_Zn_0.70_Fe(CN)_6_ as cathode and TiP_2_O_7_ as anode explored the co-insertion performance of Li^+^/K^+^ [108]. Figure 8c exhibits the schematic of the battery. Different from PBAs as Li^+^ host material, K_0.08_Ni_0.75_Zn_0.70_Fe(CN)_6_ exhibits good cyclic performance for Li^+^/K^+^ storage that can keep 63% of the original capacity after 50 cycles (Fig. 8d). It is worth noting that this system delivers an average operating voltage of 1.25 V, which is much higher than most PBAs in aqueous electrolyte. However, they did not give systematic explanation for the increase of operating voltage in this work. Later, they reported zero-strain K_0.04_Cu_1.47_Fe(CN)_6_ to co-insert Li^+^/K^+^ and explored this reason, which also exhibits a high operating potential up to 1.39 V [109]. Figure 8e exhibits the SEM image (top) and TEM image (below) of K_0.04_Cu_1.47_Fe(CN)_6_. They found that the binding energy of K^+^ inserted in its corresponding redox sites is smaller than Li^+^. Thus, K_0.04_Cu_1.47_Fe(CN)_6_ prefers to insert K^+^. When Li^+^ and K^+^ co-insert in PBAs, the presence of K^+^ will affect the insertion kinetics of Li^+^ that can shift the operation potential of PBAs to a higher value and this phenomenon can be seen in the galvanostatic profiles of the co-insertion of Li^+^/K^+^ (Fig. 8f). Obviously, the increase of cathode potential can result in the increase of battery operation voltage. For further improving the cycling performance of the mixed-ion battery, they later explored the co-insertion of Li^+^ and Na^+^ in Fe_4_[Fe(CN)_6_]_3_ [110]. Impressively, it exhibits a wonderful rate performance with no significant capacity decrease from 5 to 2 C and then retains this capacity up to 400 cycles (Fig. 8g). In summary, the co-insertion of Li^+^ with Na^+^ or K^+^ in PBAs has a broad prospect. However, there are few studies on the co-insertion of Li^+^ with other ions at present, so the future study of Li^+^ insertion behavior in PBAs can be put in this aspect.

#### ***PBAs for Aqueous K***^+^***Batteries***

**Fig. 9:**
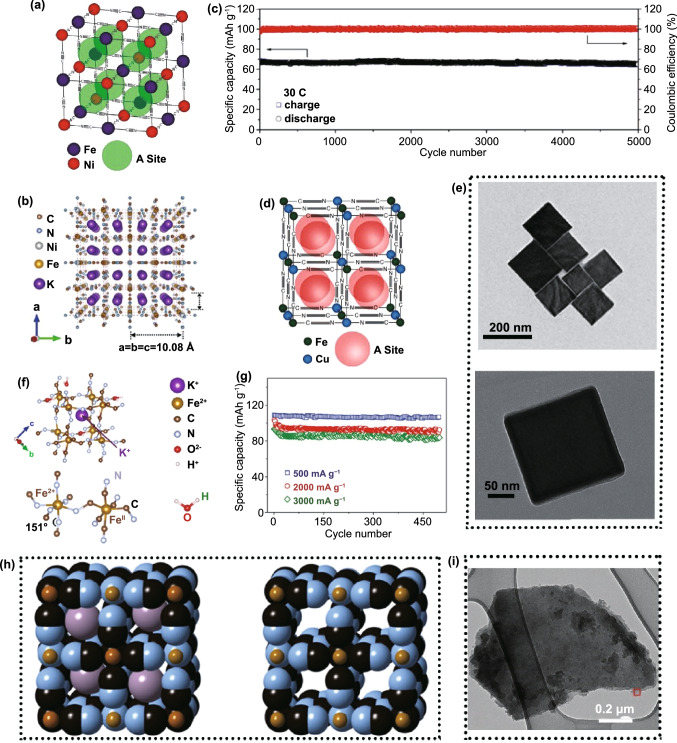
**a** Face-centered cubic structure of NiFe-PBA (K_0.6_Ni_1.2_Fe(CN)_6_·3.6H_2_O). Reproduced with permission from Ref. [43]. Copyright 2011, American Chemical Society. **b** General view of the face-centered cubic crystal structure of K_2_NiFe(CN)_6_·1.2H_2_O. **c** Long-term cycling performance at 30 C of K_2_NiFe(CN)_6_·1.2H_2_O. Reproduced with permission from Ref. [80]. Copyright 2018, Wiley–VCH. **d** Face-centered cubic structure of K_0.9_Cu_1.3_Fe(CN)_6_. Reproduced with permission from Ref. [74]. Copyright 2012, Electrochemical Society. **e** TEM image of K_2_Fe^II^[Fe^II^(CN)]_6_·2H_2_O nanocubes. **f** Refined crystal structure of FeFe-PBA. **g** Long cycle performance of K_2_Fe^II^[Fe^II^(CN)]_6_·2H_2_O nanocubes at different current density. Reproduced with permission from Ref. [7]. Copyright 2017, Wiley–VCH. **h** Crystal lattice unit cell of Prussian blue (left, KFe^III^Fe^II^(CN)_6_) and crystal lattice unit cell of Prussian green (right, Fe^III^Fe^III^(CN)_6_) (The black, blue, purple and brown balls represent C, N, K and Fe atoms, respectively). Reproduced with permission from Ref. [112]. Copyright 2015, Elsevier. **i** TEM image showing a MoS_2_ flake uniformly decorated by Prussian blue nanoparticles to form K_0.47_Fe_4_^III^[Fe^II^(CN)_6_]_3.14_@(MoSO_1.7_)_0.44_·18H_2_O. Reproduced with permission from Ref. [113]. Copyright 2018, Wiley–VCH

Due to the small hydration ion radius of K^+^ (Fig. 3b), its transport speed in the ion channel will be relatively fast. Besides, the 8c site, as the insertion site of K^+^ in PBAs, has the largest volume among all sites, which will also accelerate the transport of K^+^ in PBAs [96]. Thus, PBAs usually have an excellent rate performance in aqueous K^+^ batteries. As the crystal structure displayed in Fig. 9a, Cui and co-workers synthesized K_0.6_Ni_1.2_Fe(CN)_6_·3.6H_2_O by co-precipitation method to insert K^+^ [43]. Due to the low-strain characteristic of this K_0.6_Ni_1.2_Fe(CN)_6_·3.6H_2_O and the characteristic of K^+^ inserted in PBAs, it can retain 66% of the initial capacity even at 41.7 C and its Coulombic efficiency can reach 99% at low rates. Soon after, Zhao and co-workers synthesized nanosized potassium-rich mesoporous K_2_NiFe(CN)_6_·1.2H_2_O (Fig. 9b) through a diffuse ion-induced co-precipitation approach and constructed full cell with NaTi_2_(PO_4_)_3_ [80]. It exhibits ultrafast potassium-ion storage within 4.1 s at 500 C and 98.6% of capacity retention at 30 C after 5000 cycles (Fig. 9c). Later, Cui and co-workers proved that K_0.9_Cu_1.3_Fe(CN)_6_ (Fig. 9d) also possesses outstanding rate and cycle performance when it hosts K^+^ in aqueous electrolyte [74]. Although these PBAs both exhibit well rate performance in aqueous K^+^ batteries, they have a common shortcoming that the reversible capacity only has approximately 60 mAh g^−1^. To achieve high K-uptake capacity, Wang and co-workers synthesized high-potassium content K_2_Fe^II^[Fe^II^(CN)]_6_·2H_2_O nanocubes (Fig. 9e, f) as cathode to store K^+^ [7]. Due to the presence of two redox centers, it can exhibit a high discharge capacity of 120 mAh g^−1^ and approximately 85% capacity retention over 500 cycles at 21.4 C. Even at higher rates, it still has good electrochemical performance (Fig. 9g).

Obviously, the FeFe-PBAs used as the cathode for aqueous K^+^ batteries possess huge application potential and thus received more attention in further optimizing its electrochemical performance. The most mainstream methods are controlling the size of particle and achieving higher crystallinity. The influence of size of particle on performance can be summarized as follows: Firstly, small crystals provide shorter diffusion paths and thus diffusion rates of alkali ions are improved. Secondly, the strain produced by volume changes during ion charge and discharge processes is minimized, which in turn improves rate capability and stability. Thirdly, the increase of the surface area of PBAs can effectively improve electron transport and structural resilience [111]. Solanki and co-workers synthesized different-sized PBAs (KFe^III^Fe^II^(CN)_6_ with size of 50–75 nm, Fe^III^Fe^III^(CN)_6_ with size of 2–10 microns) as shown in Fig. 9h and explored the effect of particle size on the properties of PBAs for inserting K^+^ [112]. The results show that PBAs with small particle size do have greater advantage in electrochemical performance. For obtaining small particle size, in addition to the widely used controlled crystallization method, Coronado and co-workers propose a new method that uses MoS_2_ as reducing agent and platform to synthesize PBAs shells with small and uniform particle size (Fig. 9i) and got excellent electrochemical performance [113]. Therefore, in the future preparation of PBAs, supporting materials such as MoS_2_ can be used to provide two-dimensional active carriers to achieve uniform nucleation of nanoparticles. In general, the PBAs with open framework possess huge progress potential in aqueous K^+^ batteries and are waiting to be further explored.

#### ***PBAs for Aqueous H***^+^***Batteries***

At present, most of charge carriers of the battery under study are metal ions, and there are few researches on such carriers as H^+^. As shown in Fig. 3b, H^+^ has a radius nearly one-fifth that of Li^+^, and their hydration radius is also much smaller than that of commonly used metal charge carriers. Therefore, the insertion of H^+^ in the electrode material will result in a small lattice strain due to its smaller ionic radius [114]. In addition to smaller lattice strains, H^+^ have another advantage as carriers. In aqueous electrolyte, there are a large number of hydrogen bonds between water molecules, which form a network of hydrogen bonds. As H^+^ migrates, the H^+^ displaces hydrogen ions from one of the water molecules in the hydrogen bond network, and the displaced hydrogen ions then displace hydrogen ions from the next water molecule through the hydrogen bond, thus transferring the H^+^ to the electrode surface by continuous displacement. This mode of H^+^ transport is called Grotthuss proton conduction, and it is this mode of transport that gives the H^+^ faster migration dynamics.

For PBAs, it will produce a lot of crystal water in the crystal lattice when using the co-precipitation method. In addition, due to some defects in PBAs, these defects will expose M atoms in PBAs outside the unit cell and combine with water molecules. Then, these water molecules bonded with M atoms can communicate crystal water in each lattice and form a hydrogen bond network in PBAs. Therefore, H^+^ can also be transported by Grotthuss proton conduction in PBAs to achieve faster H^+^ transport. Combined with the small ion radius of H^+^ and the fast dynamics of Grotthuss proton conduction, PBAs as electrode for aqueous H^+^ batteries will produce excellent cyclic stability and rate performance. For the application of PBAs in aqueous H^+^ batteries, Ji et al*.* first use the Cu[Fe(CN)_6_]_0.63_∙□_0.37_∙3.4H_2_O as cathode and explore its electrochemical performance in 2.0 M H_2_SO_4_ electrolyte [81]. Impressively, the Cu[Fe(CN)_6_]_0.63_∙□_0.37_∙3.4H_2_O exhibits a capacity of 48 mAh g^−1^ at 5000 C that is the half capacity in 1 C. In addition, it shows an unprecedented long cycle performance that can retain 60% of its initial capacity at 500 C after 730,000 cycles. Later, they find that the Ni[Fe(CN)_6_]_0.60_∙□_0.40_∙4.5H_2_O also exhibits excellent cycle and rate performance when it used as electrode for aqueous H^+^ batteries [115]. Furthermore, they find that Grotthuss proton conduction can still occur even at low temperature due to the unique chemical environment of crystal water in PBAs. At -40 °C, the Ni[Fe(CN)_6_]_0.60_∙□_0.40_∙4.5H_2_O still possesses well rate performance that can deliver ∼50% of theoretical capacity at 50 C. Therefore, the application of PBAs in aqueous H^+^ batteries at low temperature also has a broad development prospect.

#### ***PBAs for Aqueous NH***_***4***_^+^***Batteries***

Apart from H^+^, NH_4_^+^ as a non-metallic charge carrier also has received more and more attention in recent years. In 2011, Cui and co-workers explored the insertion performance of NH_4_^+^, Na^+^ and K^+^ in K_0.9_Cu_1.3_Fe(CN)_6_ and K_0.6_Ni_1.2_Fe(CN)_6_ [74]. Due to the large ionic radius of NH_4_^+^, it exhibits a higher insertion potential. Furthermore, both of the two PBAs exhibit excellent cycle stability during the insertion of NH_4_^+^, which opens the door of the PBAs in aqueous NH_4_^+^ batteries. The first rocking-chair NH_4_^+^ battery was proposed by Ji and co-workers [79]. For this battery, it uses (NH_4_)_1.47_Ni[Fe(CN)_6_]_0.88_ as cathode and 3,4,9,10-perylenetetracarboxylic diimide (PTCDI) as anode. Due to the good structure stability of (NH4)_1.47_Ni[Fe(CN)_6_]_0.88_, it exhibits an initial capacity of 51.3 mAh g^−1^ and retains 74% at 5 C after 2000 cycles. However, due to the limited stability of the anode, the cycling stability of this full cell is worse that can retain 67% at 3 C after 1000 cycles. Later, Huang *et.al.* proposed a novel aqueous Zn^2+^/NH_4_^+^ hybrid battery that uses the K_0.72_Cu[Fe(CN)_6_]_0.78_·3.7H_2_O as cathode, zinc as anode in 1 M (NH_4_)_2_SO_4_/0.1 M ZnSO_4_ electrolyte [116]. It is noteworthy that the insertion/extraction of NH_4_^+^ and deposition/plating of Zn^2+^ occurs in cathode and anode, respectively, during the battery working. The K_0.72_Cu[Fe(CN)_6_]_0.78_·3.7H_2_O shows an excellent cyclic stability that can retain 78% at the current density of 1800 mA g^−1^ after 1000 cycles. Impressively, due to the stability of the zinc anode in the electrolyte, the full cell shows a better cycle stability that can retain 76.5% at the current density of 1800 mA g^−1^ after 1000 cycles. Thus, the zinc anode is worthy of consideration in future studies of PBAs in aqueous NH_4_^+^ batteries. Aside from the CuFe-PBAs and NiFe-PBAs, the FeFe-PB (Fe[Fe(CN)_6_]_0.88_·□_0.12_·2.8H_2_O) in aqueous NH_4_^+^ battery also has well electrochemical performance [117]. Impressively, the Fe[Fe(CN)_6_]_0.88_·□_0.12_·2.8H_2_O exhibits a nearly zero strain characteristic when it hosts NH_4_^+^. Thus, its cyclic retention rate can reach up to 78% after 50,000 cycles. By increasing the agitation rate during the preparation of PBAs, Huang and co-workers synthesized NaFe^III^Fe^II^(CN)_6_ with ball-cutting morphology [118]. Compared with the cubic morphology NaFe^III^Fe^II^(CN)_6_, the ball-cutting morphology NaFe^III^Fe^II^(CN)_6_ improves the uniformity and reduces the particle size of NaFe^III^Fe^II^(CN)_6_. So, it possesses an excellent cycle performance with no fading after 50,000 cycles. In conclusion, different types of PBAs both have good electrochemical performance in hosting NH_4_^+^ and waiting to be further explored.

### PBAs for Aqueous Multivalent-Ion Batteries

For the storage of multivalent ions, it mainly presents three challenges. Firstly, multivalent ion possesses strong hydration in the process of ion diffusion [119]. After hydration, the hydrated ionic radius of multivalent ion is going to be particularly large and the specific change value is shown in Fig. 3b. Secondly, the insertion of multivalent ion in electrode needs more electrons to retain electric neutrality, which means more valence changes will occur. Generally speaking, the large sudden change of valence will cause large local deformation to the crystal structure of host materials [120–122]. Thirdly, the multivalent ion will be subject to stronger electrostatic repulsion in host materials that will result in the instability of crystal structure. Therefore, the selection of electrode materials for aqueous multivalent-ion batteries becomes particularly important.

For PBAs electrode materials, it has a large ion insertion site which can host the multivalent ion with large hydrated ionic radius. Besides, the vacancy with large volume in PBAs structure also can be used as insertion site for multivalent ion in aqueous electrolyte. As shown in Fig. 2, in the crystal structure of PBAs, the vacancy site often absorbs some coordinated water molecules. Therefore, compared with other insertion site, the vacancy site will allow the host ions adjacent to more coordinated water molecules within the structure, thus forming a hydration shell. This hydration shell can provide more electrostatic shielding to host ions and thus improve the thermodynamic stability of host ions at vacancy sites. It is this mechanism that allows vacancies to be used as insertion sites for multivalent ions. Meanwhile, the PBAs possess multiple redox sites, which make them able to accept more valence changes. Combined with these advantages, PBAs will be a highly potential electrode material for aqueous multivalent-ion batteries. Next, these sections will detail the applications of different PBAs in various aqueous multivalent-ion batteries.

#### ***PBAs for Aqueous Mg***^***2***+^***Batteries***

**Fig. 10:**
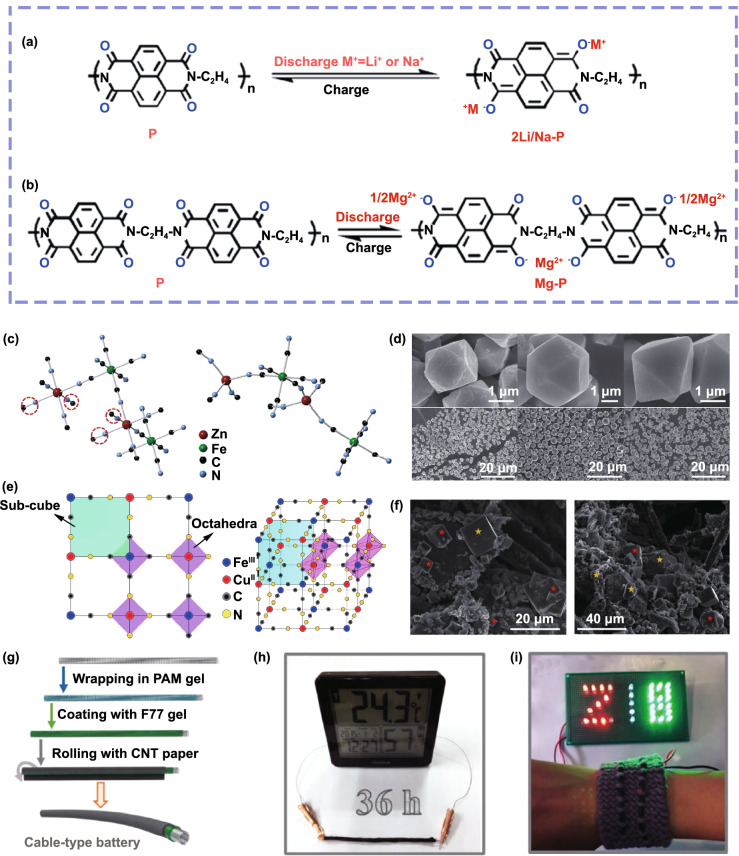
**a** Electrochemical redox mechanism (insertion and extraction) of polyimide with Li^+^ and Na^+^. **b** Electrochemical redox mechanism (insertion and extraction) of polyimide with Mg^2+^. Reproduced with permission from Ref. [124]. Copyright 2017, American Chemical Society. **c** Coordination environments for Zn and Fe atoms in cubic structure (left) and rhombohedral structure (right) of Zn_3_[Fe(CN)_6_]_2_. **d** SEM and TEM images of cuboctahedron, truncated octahedral and octahedral Zn_3_[Fe(CN)_6_]_2_. Reproduced with permission from Ref. [17]. Copyright 2015, Nature publishing Group. **e** Crystal structure of KCuFe(CN)_6_ nanocube. Reproduced with permission from Ref. [125]. Copyright 2015, Elsevier. **f** SEM images of CuZnHCF (93:7). Reproduced with permission from Ref. [129]. Copyright 2019, Elsevier. **g** Schematic illustration for the fabrication of the cable-type Zn/CoFe(CN)_6_ battery. **h** An electronic hygrometer powered by a cable-type battery. **i** A textile wristband integrated with two cable batteries connected in series as power accessory for different color LEDs. Reproduced with permission from Ref. [77]. Copyright 2019, Wiley–VCH

Magnesium is one of the largest reserves of metals in nature, which makes Mg^2+^ batteries have a greater cost advantage over Li^+^ batteries. In addition, aqueous Mg^2+^ batteries also show great advantages in terms of safety and environmental friendliness. Due to its excellent performance in Na^+^ and K^+^ batteries, PBAs have been promoted to be applied in aqueous Mg^2+^ batteries in recent years. In 2013, Cui et al*.* systematically explored the electrochemical behavior of Mg^2+^ storage in K_0.6_Ni_1.2_Fe(CN)_6_ cathode and proposed a novel solution to decrease capacity decay [75]. For the insertion of Mg^2+^ in K_0.6_Ni_1.2_Fe(CN)_6_, they found that the partial dissolution of K_0.6_Ni_1.2_Fe(CN)_6_ in the electrolyte caused significant decay process at the beginning of the cycle and then stabilization. By adding a small amount of Ni^2+^ to the electrolyte, not only the capacity is recovered but also the cycling stability is improved. The principle of this method can be attributed to two points: Firstly, in the process of discharge, Ni^2+^ in the electrolyte is inserted into PBAs to improve the structural stability of PBAs; secondly, electrolyte with a certain concentration of Ni^2+^ can inhibit the dissolution of high-spin Ni atom. Obviously, this simple method of improving PBAs cycling performance by changing electrolyte composition deserves more attention. For CuFe-PBAs, Yamada et al*.* reported the reversible insertion of Mg^2+^ in K_0.1_Cu[Fe(CN)_6_]_0.7_·3.6H_2_O [123]. Impressively, through ex situ Mössbauer spectroscopy, they reveal that both Fe^3+^/Fe^2+^ and Cu^II^/Cu^I^ experience solid-state redox when Mg^2+^ inserts into K_0.1_Cu[Fe(CN)_6_]_0.7_·3.6H_2_O. About the PBAs in aqueous full Mg^2+^ batteries, Xia and co-workers recently used Na_1.4_Ni_1.3_Fe(CN)_6_·5H_2_O cathode and polyimide anode to construct a full battery [124]. Figure 10a, b shows the electrochemical redox mechanism (insertion and extraction) of polyimide with Li^+^, Na^+^ and Mg^2+^. For this battery, it exhibits a high output voltage of 1.5 V and an excellent cycle life that can retain about 60% of its initial capacity after 5000 cycles, which is currently the best cycling performance of aqueous Mg^2+^ battery. Obviously, it has a strong guiding value for the future study for the PBAs used in aqueous full Mg^2+^ batteries.

#### ***PBAs for Aqueous Zn***^***2***+^***Batteries***

About the PBAs in aqueous Zn^2+^ batteries, Liu et al*.* proved that K_2_Zn_3_[Fe(CN)_6_]_2_ is more suitable to be used in aqueous Zn^2+^ batteries than aqueous Na^+^ and K^+^ batteries because K_2_Zn_3_[Fe(CN)_6_]_2_ has better structural stability in aqueous Zn^2+^ electrolyte [22]. Besides, K_2_Zn_3_[Fe(CN)_6_]_2_-based full battery was successfully assembled the Zn anode, achieving a high output voltage of 1.7 V and an energy density of 100 Wh kg^−1^. Later, they explored the effect of Zn_3_[Fe(CN)_6_]_2_ with different morphologies for Zn^2+^ insertion [17]. Figure 10c shows the coordination environments for Zn and Fe atoms in two different crystal systems (cubic and rhombohedral), and corresponding SEM images of three Zn_3_[Fe(CN)_6_]_2_ with different morphologies are shown in Fig. 10d. And the experiment results show that, compared with truncated octahedral and octahedral particles, cube octahedral particles have more advantages in rate and cycling performance because the cube octahedral structure surface orientations is more beneficial to Zn^2+^ diffusion and Zn_3_[Fe(CN)_6_]_2_ structure stability.

About the CuFe-PBAs in aqueous Zn^2+^ battery, Wang and co-workers demonstrated for the first time that Zn^2+^ can reversibly insert/extract into/from KCuFe(CN)_6_ nanocube (Fig. 10e) in 1 M ZnSO_4_ electrolyte [125]. However, its cycling stability is poor with 77% capacity retention after 20 cycles. For comparison, Mantia and co-workers reported that KCuFe(CN)_6_ (K_0.71_Cu[Fe(CN)_6_]_0.72_·3.7H_2_O) can provide 90% of theoretical capacity with a capacity retention of 96.3% after 100 cycles at 1 C in a dilute electrolyte of 20 mM ZnSO_4_ solution [126]. According to the conventional view, the aging performance may be attributed to the destruction of crystal structure of PBAs upon Zn^2+^ insertion/extraction. However, Mantia and co-workers failed to find Cu and Fe elements in the electrolyte through ICP-MS analysis [127]. For this phenomenon, Lim and co-workers claimed that the inserted Zn^2+^ not only existed in the interstitial lattice vacancy but also appeared in the pre-occupied lattice site during the cycling process [128]. Zn atoms in the crystal structure will replace Cu atoms, leading to the formation of Cu(CN)_2_ and CuZn(CN)_4_. Since Cu(CN)_2_ and CuZn(CN)_4_ do not have redox couple, they cannot provide capacity. Thus, this irreversible phase transition leads to the capacity fading. In particular, the lower the concentration of Zn^2+^ in the solution, the more difficult this irreversible phase transition is to occur, which explains why KCuFe(CN)_6_ has better performance in 20 mM ZnSO_4_ electrolyte compared with 1 M ZnSO_4_ electrolyte. In order to overcome this irreversible phase change, Mantia fabricated a series of copper zinc hexacyanoferrates (CuZnHCF) mixtures with different Cu/Zn ratios [129]. It is found that the irreversible phase transition can be effectively avoided for CuZnHCF with Cu/Zn ratio of 93:7 (SEM image shown in Fig. 10f) and the capacity fading problem can be solved to a large extent. Compared with the CuHCF without Zn element, it exhibits better capacity retention of 85.5% after 1000 cycles in 20 mM ZnSO_4_ solution. Obviously, this method provides a new direction for optimizing PBAs in aqueous multivalent-ion batteries. About the application of double-atom redox PBAs in aqueous Zn^2+^ batteries, Zhi and co-workers synthesized KCoFe(CN)_6_ and used it as cathode of aqueous Zn^2+^ batteries [77]. With two redox sites, this battery exhibits a high capacity of 173.4 mAh g^−1^ at current density of 0.3 A g^−1^. Impressively, they adopt a sol–gel transition strategy constructing a solid-state cable-type battery, and Fig. 10g schematically illustrates its configuration. Figure 10h, i shows the practical application of the solid-state cable-type battery in two different electronic devices, electronic hygrometer and LEDs, respectively. Obviously, combined with the lower electrode potential of zinc anode, the cable-type battery based on double-atom redox PBAs exhibits a huge potential in flexible energy storage that provides a new direction for the practical application of aqueous battery based on PBAs.

#### ***PBAs for Aqueous Ca***^***2***+^***Batteries***

Since hydrated Ca^2+^ binds fewer water molecules on the surface [130], the investigations of aqueous Ca^2+^ batteries have great development potential. About the PBAs used in aqueous Ca^2+^ batteries, Yao and co-workers synthesized K_0.02_Cu[Fe(CN)_6_]_0.66_⋅3.7H_2_O as cathode material for Ca^2+^ storage [131]. Due to the low crystal structure strain (1.1%), the K_0.02_Cu[Fe(CN)_6_]_0.66_⋅3.7H_2_O exhibits an excellent long cycle performance that can retain 88% of its initial capacity at 5 C after 2000 cycles. In addition, the K_0.02_Cu[Fe(CN)_6_]_0.66_⋅3.7H_2_O also exhibits excellent rate performance in aqueous Ca^2+^ batteries. Its capacity can be retained to 65% when the current density increases from 0.2 C to 20 C, while that of in aqueous Mg^2+^ battery can only be maintained to 33%. This difference in rate performance can be summarized in two points: First, Ca^2+^ with a smaller charge density will encounter less resistance when it is inserted/extracted into/from the PBAs. Second, hydrated Ca^2+^ binds fewer water molecules on the surface, speeding up the transport of Ca^2+^in PBAs. Similarly, the K_0.02_Cu[Fe(CN)_6_]_0.64_·9H_2_O also demonstrated same excellent Ca^2+^ storage performance in aqueous Ca^2+^ battery, which can retain 94% of its initial capacity at 5 C after 1000 cycles [132].

About the electrochemical performance of PBAs in aqueous Ca^2+^ battery, the concentration of electrolyte also has influence. Jeong and co-workers studied the electrochemical properties of CuFe(CN)_6_ in the different concentrations of electrolyte and found that in 1.0 mol dm^−3^ Ca(NO_3_)_2_, the CuFe(CN)_6_ delivers a 60 mAh g^−1^ initial capacity and retains 50% at 10 C after 1000 cycles [133]. But in 8.4 dm^−3^ Ca(NO_3_)_2_, it exhibits a better storage performance that can deliver a 70 mAh g^−1^ initial capacity and retains 95% at 10 C after 1000 cycles because the hydration number of Ca^2+^ in the high-concentration electrolyte was smaller. Therefore, it is a simple and effective method to improve the electrochemical performance of PBAs in aqueous Ca^2+^ batteries by changing the electrolyte concentration, which is worthy to be applied in future studies on aqueous Ca^2+^ batteries.

#### ***PBAs for Aqueous Al***^***3***+^***Batteries***

**Fig. 11:**
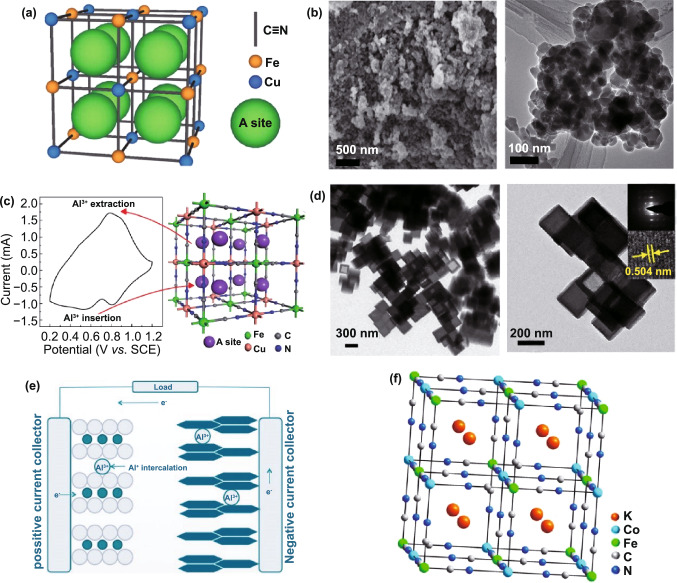
**a** Structure of K_0.03_Cu[Fe(CN)_6_]_0.65_·2.6H_2_O and corresponding insertion sites for trivalent ions. Reproduced with permission from Ref. [76]. Copyright 2015, Wiley–VCH. **b** SEM and TEM images of as-prepared KCu[Fe(CN)_6_]·8H_2_O powders. **c** Typical CV curve of KCu[Fe(CN)_6_]·8H_2_O in Al_2_(SO_4_)_3_ solution and the schematic positions of Al^3+^ in the framework. Reproduced with permission from Ref. [10]. Copyright 2015, The Royal Society of Chemistry. **d** TEM image of K_2_CoFe(CN)_6_. Reproduced with permission from Ref. [135]. Copyright 2020, Elsevier. **e** Working process of aqueous CuFe(CN)_6_/TiO_2_ battery for Al^3+^ storage. Reproduced with permission from Ref. [134]. Copyright 2018, Springer. **f** Crystal structure of K_2_CoFe(CN)_6_. Reproduced with permission from Ref. [135]. Copyright 2020, Elsevier

For the insertion of Al^3+^ in CuFe-PBAs, Cui et al. use K_0.03_Cu[Fe(CN)_6_]_0.65_·2.6H_2_O as host material to reversibly store Al^3+^ [76]. Figure 11a presents the lattice structure with all occupied atoms and possible insertion sites for hosting ions in this K_0.03_Cu[Fe(CN)_6_]_0.65_·2.6H_2_O. Furthermore, they confirmed the shielding effect of crystal water and hydration shell of Al^3+^ on electrostatic repulsion between Al^3+^ and K_0.03_Cu[Fe(CN)_6_]_0.65_·2.6H_2_O, which lays a foundation for the insertion of Al^3+^ in PBAs. Later, Gao et al*.* used Al_2_(SO_4_)_3_ solution as aqueous electrolyte to explore the Al^3+^ reversible insertion characteristic in nanosized KCu[Fe(CN)_6_]·8H_2_O (Fig. 11b) [10]. As reported in Fig. 11c, typical redox couples in CV curve are well indexed to the schematic insertion/extraction positions of Al^3+^ in PBAs framework. For Al^3+^ uptake, KCu[Fe(CN)_6_]·8H_2_O can release a discharge capacity of 46.9 mAh g^−1^ at 400 mA g^−1^ with retention of 54.9% after 1000 cycles. In addition to reducing electrostatic repulsion, PBAs with small particle size can also improve the migration rate of Al^3+^ by reducing the diffusion distance. This is proved by Wills and Pang et al. via the investigation on CuFe(CN)_6_ (Fig. 11e) and K_2_CoFe(CN)_6_ (Fig. 11d, f), respectively, and got well performance [134, 135]. Although these two kinds of PBAs have wonderful Al^3+^ insertion ability, the reason of capacity fading is not clear. Later, Wu and co-workers reported the working mechanism in PBAs-based Al^3+^ batteries [136]. They clarified that the reversible insertion/extraction of Al^3+^ in K_0.02_Ni_1.45_[Fe(CN)_6_]·2.6H_2_O is related to a single-phase reaction without producing polycrystalline structure. In addition, they also found that the capacity fading is not mainly caused by the collapse of PBAs structure, but the transfer of Ni in K_0.02_Ni_1.45_[Fe(CN)_6_]·2.6H_2_O to Al foil anode, resulting in interface instability between anode and electrolyte. Obviously, this study provides a new idea for us to solve capacity fading problem of PBAs upon multivalent ions uptake.

#### PBAs for Other Aqueous Multivalent Metal-Ion Batteries

Besides these above multivalent metal ions, the insertion performance of Ba^2+^, Sr^2+^, Fe^2+^, Co^2+^, Ni^2+^, Cu^2+^, Pb^2+^, Y^3+^, La^3+^, Ce^3+^, Nd^3+^ and Sm^3+^ in PBAs also has been explored. For Ba^2+^ and Sr^2+^, Cui and co-workers explored their insertion performance in NiFe(CN)_6_ [75]. When NiFe(CN)_6_ inserting Sr^2+^, it exhibits an initial capacity of 40 mAh g^−1^ and retains 64% at 5 C after 2000 cycles. Because Ba^2+^ has smaller hydrated ionic radius, the insertion performance of NiFe(CN)_6_ in aqueous Ba^2+^ battery is better. It exhibits a 42 mAh g^−1^ initial capacity and retains 93% at 5 C after 2000 cycles. About these two ions as host ion in PBAs, the studies are few. Thus, future studies can put into these aspects. Later, Cui et al*.* continue to explore the insertion performance of Co^2+^, Ni^2+^, Cu^2+^, Pb^2+^, Y^3+^, La^3+^, Ce^3+^, Nd^3+^ and Sm^3+^ in K_0.03_Cu[Fe(CN)_6_]_0.65_·2.6H_2_O [76]. They find that K_0.03_Cu[Fe(CN)_6_]_0.65_·2.6H_2_O has a serious decay process when inserting Co^2+^, Ni^2+^ and Cu^2+^ resulting in poor cycling performance. The main reason for this phenomenon is that partial replacement of these ions with the high-spin transition metal atom in K_0.03_Cu[Fe(CN)_6_]_0.65_·2.6H_2_O results in irreversible phase transition. Therefore, the main problem for the insertion of these ions into PBAs is to resolve this irreversible phase transition. It should be noted that the insertion and extraction of Fe^2+^ in FeFe-PB do not produce this irreversible structural phase change. In 2019, Ji et al*.* used Fe[Fe(CN)_6_]_0.73_∙□_0.27_∙3.6H_2_O as cathode, Fe as anode and FeSO_4_ solution as electrolyte to build a full cell [137]. For this cell, it exhibits an excellent cycle stability that can retain 88% of its initial capacity at 1 C after 1000 cycles.

In addition to these common metal ions, Cui et al*.* found that some lanthanide metal ions can also be reversibly inserted/extracted in/from PBAs such as La^3+^, Ce^3+^, Nd^3+^ and Sm^3+^. For these ions inserted into K_0.03_Cu[Fe(CN)_6_]_0.65_·2.6H_2_O, they all show similar voltammogram, which means that they have the same insertion sites in K_0.03_Cu[Fe(CN)_6_]_0.65_·2.6H_2_O. Impressively, they both can deliver a capacity of approximately 60 mAh g^−1^ at 0.2 C. Although these lanthanide metal ions can be reversibly inserted/extracted in/from PBAs, they are rarely studied due to their low natural reserves and high cost. It is worth noting that the insertion of Pb^2+^ and Y^3+^ possesses the fastest ion migration kinetics and most reversible electrochemistry among these uncommon divalent and trivalent metal ions, respectively [76]. For the insertion of Pb^2+^ in K_0.03_Cu[Fe(CN)_6_]_0.65_·2.6H_2_O, it exhibits an initial capacity of 55 mAh g^−1^ and retains 73% at 5 C after 2000 cycles. When the K_0.03_Cu[Fe(CN)_6_]_0.65_·2.6H_2_O inserting Y^3+^, it exhibits an initial capacity of 40 mAh g^−1^ and retains 87% at 5 C after 2000 cycles. Obviously, the insertion of these two ions in PBAs possesses great exploration value and is waiting to be studied.

### Relationship between Performance and Structure

**Fig. 12:**
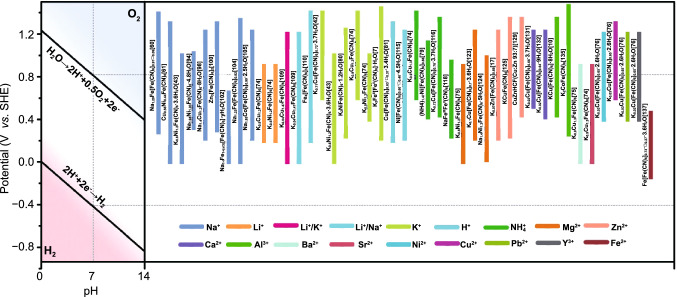
Voltage working window of PBAs for various aqueous batteries

From the above introduction, it can be clearly found that for PBAs, their electrochemical performance (redox potential, capacity, cycling stability and rate performance) is closely related to its structure (metal atom in high-spin state, defect, particle size, morphology, etc*.*) and these electrochemical performances of various PBAs in aqueous battery are summarized in Table 1. From Table 1, it can be concluded that different types of metal in high-spin state lead to different redox potential and voltage platforms for PBAs. When Fe, Co and Mn are in high-spin state, this type of PBAs has two voltage platforms because both these metals and Fe in low spin have electrochemical activity. Conversely, when Ni, Cu and Zn are in high-spin state, this type of PBAs has only one voltage platform because the Ni, Cu and Zn are electrochemically inert. It is worth noting that Cu is sometimes electrochemically active in some acidic electrolytes and produces voltage platform. Meanwhile, in terms of redox potential, FeFe-PB, CoFe-PBAs and MnFe-PBAs are generally higher than NiFe-PBAs, CuFe-PBAs and ZnFe-PBAs. Although the redox potential of different PBAs is different in aqueous batteries, most of them are within the decomposition voltage of water (Fig. 12). A small part of PBAs whose charging potential exceeds the oxygen evolution potential can be still used as electrode in aqueous battery by increasing electrolyte concentration or adjusting pH to inhibit the evolution of oxygen.

**Table 1:** Electrochemical performance of representative PBAs in various aqueous batteries

Sample	Electrolyte	Redox potential (V)	Rate capability (mAh g^−1^)	Reversible capacity (mAh g^−1^)	Cycling stability	Refs.
Na_1.29_Fe[Fe(CN)_6_]_0.91_□_0.09_	0.5 M Na_2_SO_4_	0.35/1.34	40 at 5 A g^−1^	107 at 500 mA g^−1^	100%@0.5Ag^−1^ after1100 cycles	[60]
Cu_0.56_Ni_0.44_Fe(CN)_6_	1.0 M NaNO_3_	0.78	N.A	53 at 0.8 C	100%@8 C after 2000 cycles	[61]
Na_2_VO_x_[Fe(CN)_6_]	3.0 M NaNO_3_	0.59/0.96	54 at 38.7 C	91 at 110 mA g^−1^	61%@1.2 C after 250 cycles	[69]
K_0.6_Ni_1.2_Fe(CN)_6_∙3.6H_2_O	1.0 M NaNO_3_	0.6	39 at 41.7 C	59 at 0.83 C	91%@8.3 C after 5000 cycles	[43]
K_0.9_Cu_1.3_Fe(CN)_6_	1.0 M NaNO_3_	0.78	20 at 41.7 C	58 at 0.83 C	80%@8.3 C after 500 cycles	[74]
K_0.6_Ni_1.2_Fe(CN)_6_	1.0 M NaNO_3_	0.58	40 at 41.7 C	57 at 0.83 C	91%@8.3 C after 500 cycles	[74]
Na_1.94_Ni_1.03_Fe(CN)_6_·4.8H_2_O	1.0 M Na_2_SO_4_	0.62	61 at 10 C	65 at 1 C	88%@5 C after 500 cycles	[94]
Na_1.4_Cu_1.3_Fe(CN)_6_·8H_2_O	1.0 M Na_2_SO_4_	0.8	38 at 100 C	58 at 5 C	94%@5 C after 500 cycles	[98]
Zn_3_[Fe(CN)_6_]_2_	1.0 M NaClO_4_/PEG-400	1.12	34 at 20 C	66 at 1 C	92%@5 C after 200 cycles	[100]
Na_1-x_Fe_1+(x/3)_[Fe(CN)_6_]·yH_2_O	1.0 M Na_2_SO_4_	N.A	25 at 10 C	65 at 0.2 C	80%@1 C after 200 cycles	[102]
Na_1.33_Fe[Fe(CN)_6_]_0.82_	1.0 M Na_2_SO_4_	0.32/1.31	102 at 20 C	125 at 2 C	80%@10 C after 500 cycles	[104]
Na_1.85_Co[Fe(CN)_6_]_0.99_·2.5H_2_O	1.0 M Na_2_SO_4_	0.62/1.12	60 at 20 C	130 at 1 C	90%@5 C after 800 cycles	[105]
Na_1.24_Mn[Fe(CN)_6_]_0.81_∙1.28H_2_O	10 M NaClO_4_	0.81/1.4	98 at 5.0 mA cm^−2^	116 at 120 mA g^−1^	72%@2.0 mAcm^−2^ after100cycls	[107]
K_0.9_Cu_1.3_Fe(CN)_6_	1.0 M LiNO_3_	0.6	38 at 41.7 C	55 at 0.83 C	38%@8.3 C after 500 cycles	[74]
K_0.6_Ni_1.2_Fe(CN)_6_	1.0 M LiNO_3_	0.38	35 at 41.7 C	58 at 0.83 C	40%@8.3 C after 500 cycles	[74]
K_0.08_Ni_0.75_Zn_0.70_Fe(CN)_6_	0.1 M Li_2_SO_4_/0.4 M K_2_SO_4_	0.82	N.A	60 at 1 C	63%@1 C after 50 cycles	[108]
K_0.04_Cu_1.47_Fe(CN)_6_	0.1 M Li_2_SO_4_/0.4 M K_2_SO_4_	0.9	51 at 60 C	59 at 1 C	80%@1.5 C after 100 cycles	[109]
K_0.04_Cu_1.47_Fe(CN)_6_	0.25 M Li_2_SO_4_/0.25 M Na_2_SO_4_	0.8	46 at 60 C	60 at 1 C	N.A	[109]
Fe_4_[Fe(CN)_6_]_3_	0.1 M LiClO_4_/0.4 M NaClO_4_	0.44/1.44	90 at 10 C	125 at 1 C	N.A	[110]
K_0.71_Cu[Fe(CN)_6_]_0.72_∙3.7H_2_O	1.0 M KNO_3_	0.94	40 at 83 C	59 at 0.83 C	94.6%@17 C after 10,000 cycles	[42]
K_0.6_Ni_1.2_Fe(CN)_6_∙3.6H_2_O	1.0 M KNO_3_	0.7	39 at 41.7 C	58 at 0.83 C	95%@8.3 C after 5000 cycles	[43]
K_2_NiFe(CN)_6_·1.2H_2_O	1.0 M KNO_3_	0.61	42 at 500 C	77 at 5 C	98%@30 C after 5000 cycles	[80]
K_0.9_Cu_1.3_Fe(CN)_6_	1.0 M KNO_3_	0.9	50 at 41.7 C	59 at 0.83 C	99%@8.3 C after 5000 cycles	[74]
K_0.6_Ni_1.2_Fe(CN)_6_	1.0 M KNO_3_	0.7	39 at 41.7 C	57 at 0.83 C	100%@8.3 C after 5000 cycles	[74]
K_2_Fe^II^[Fe^II^(CN)]_6_·2H_2_O	0.5 M K_2_SO_4_	0.5/1.14	88 at 21.4 C	118 at 1.4 C	96%@3.6 C after 500 cycles	[7]
Cu[Fe(CN)_6_]_0.63_∙□_0.37_∙3.4H_2_O	2.0 M H_2_SO_4_	0.45/0.8	49 at 4000 C	95 at 1 C	60%@500 C after 730,000 cycles	[81]
Ni[Fe(CN)_6_]_0.60_∙□_0.40_∙4.5H_2_O	1.0 M H_2_SO_4_	0.77	39 at 4000 C	65 at 1.5 C	73%@10 C after 1000 cycles	[115]
K_0.9_Cu_1.3_Fe(CN)_6_	0.5 M (NH_4_)_2_SO_4_	1.05	45 at 41.7 C	60 at 0.83 C	91%@8.3 C after 500 cycles	[74]
K_0.6_Ni_1.2_Fe(CN)_6_	0.5 M (NH_4_)_2_SO_4_	0.8	21 at 41.7 C	50 at 0.83 C	88%@8.3 C after 500 cycles	[74]
(NH_4_)_1.47_Ni[Fe(CN)_6_]_0.88_	1.0 M (NH_4_)_2_SO_4_	0.85	22 at 30 C	60 at 2.5 C	74%@5 C after 2000 cycles	[79]
K_0.72_Cu[Fe(CN)_6_]_0.78_·3.7H_2_O	1.0 M (NH_4_)_2_SO_4_	0.97	48.5 at 2.4 A g^−1^	58.8 at 0.3 A g^−1^	78.4%@1.8 A g^−1^ after 1000 cycles	[116]
Fe[Fe(CN)_6_]_0.88_·□_0.12_·2.8H_2_O	0.5 M (NH_4_)_2_SO_4_	0.5/0.7	48 at 8.8 C	90 at 1.1 C	88%@2.2 C after 450 cycles	[117]
NaFe^III^Fe^II^(CN)_6_	1.0 M (NH_4_)_2_SO_4_	0.47/0.83	48 at2 A g^−1^	62 at 0.25 A g^−1^	100%@2 A g^−1^ after 50,000 cycles	[118]
K_0.6_Ni_1.2_Fe(CN)_6_	1.0 M Mg(NO_3_)_2_	0.4	47 at 0.2 C	31 at 10 C	65%@5 C after 2000 cycles	[75]
K_0.1_Cu[Fe(CN)_6_]_0.7_·3.6H_2_O	1.0 M Mg(NO_3_)_2_	0.82	37 at 1 A g^−1^	50 at 0.1 A g^−1^	N.A	[123]
Na_1.4_Ni_1.3_Fe(CN)_6_·5H_2_O	1.0 M MgSO_4_	0.72	39 at 10 A g^−1^	65 at 0.1 A g^−1^	94.8%@1 A g^−1^ after 2000 cycles	[124]
K_2_Zn_3_[Fe(CN)_6_]_2_	1.0 M ZnSO_4_	1.12	32.3 at 20 C	65.4 at 1 C	81%@5 C after 100 cycles	[22]
K_0.07_Zn[Fe(CN)_6_]_0.69_	3.0 M ZnSO_4_	1.1	60.5 at 3 A g^−1^	69.1 at 0.06 A g^−1^	93%@0.3 A g^−1^ after 100 cycles	[17]
K_0.08_Zn[Fe(CN)_6_]_0.67_	3.0 M ZnSO_4_	1.1	50.3 at 3 A g^−1^	67.3 at 0.06 A g^−1^	86.6%@0.3 A g^−1^ after 100 cycles	[17]
K_0.07_Zn[Fe(CN)_6_]_0.68_	3.0 M ZnSO_4_	1.1	36 at 3 A g^−1^	66 at 0.06 A g^−1^	72%@0.3 A g^−1^ after 100 cycles	[17]
KCuFe(CN)_6_	1.0 M ZnSO_4_	0.94	N.A	56 at 0.02 A g^−1^	77%@0.02 A g^−1^ after 20 cycles	[125]
K_1+2y_Cu_1-y_V_y_[Fe(CN)_6_]_z_	20 mM ZnSO_4_	0.84	49 at 10 C	58 at 1 C	78%@10C after 1000 cycles	[127]
CuZnHCF (Cu/Zn = 93:7)	20 mM ZnSO_4_	0.9	N.A	50 at 1 C	98.12%@1 C after 500 cycles	[129]
CoFe(CN)_6_	4.0 M Zn(OTf)_2_	0.55/0.89	173.4 at 6 A g^−1^	173.4 at 0.3 A g^−1^	100%@3 A g^−1^ after 2200 cycles	[77]
K_0.02_Cu[Fe(CN)_6_]_0.66_⋅3.7H_2_O	2.5 M Ca(NO_3_)_2_	0.96	37 at 20 C	58 at 0.2 C	88%@5 C after 2000 cycles	[131]
K_0.02_Cu[Fe(CN)_6]0.64_·9H_2_O	2.5 M Ca(NO_3_)_2_	0.84	51 at 5 C	60 at 1 C	94%@5 C after 1000 cycles	[132]
CuFe(CN)_6_	8.4 M Ca(NO_3_)_2_	0.82	61 at 10 C	66 at 0.2 C	88.6%@10 C after 5000 cycles	[133]
K_0.03_Cu[Fe(CN)_6_]_0.65_·2.6H_2_O	1.0 M Al(NO_3_)_3_	0.7	43 at 10 C	62 at 0.2 C	N.A	[76]
KCu[Fe(CN)_6_]·8H_2_O	0.5 M Al(SO_4_)_3_	0.83	46.9 at 0.4 A g^−1^	62.9 at 0.05 A g^−1^	54.9%@0.4 A g^−1^ after 1000 cycles	[10]
K_2_CoFe(CN)_6_	1.0 M Al(NO_3_)_3_	0.75/0.95	18 at 1 A g^−1^	50 at 0.1 A g^−1^	76%@0.1 A g^−1^ after 1600 cycles	[135]
K_0.02_Ni_1.45_[Fe(CN)_6_]·2.6H_2_O	0.5 M Al(SO_4_)_3_	0.78	N.A	47 at 0.02 A g^−1^	53%@0.02 A g^−1^ after 500 cycles	[136]
K_0.6_Ni_1.2_Fe(CN)_6_	0.1 M Ba(NO_3_)_2_	0.5	30 at 10 C	50 at 0.2 C	85%@5 C after 2000 cycles	[75]
K_0.6_Ni_1.2_Fe(CN)_6_	1.0 M Sr(NO_3_)_2_	0.45	38 at 10 C	51 at 0.2 C	79%@5 C after 2000 cycles	[75]
K_0.03_Cu[Fe(CN)_6_]_0.65_·2.6H_2_O	1.0 M Ni(NO_3_)_2_	0.82	24 at 0.2 C	57 at 0.2 C	50%@5 C after 2000 cycles	[76]
K_0.03_Cu[Fe(CN)_6_]_0.65_·2.6H_2_O	1.0 M Cu(NO_3_)_2_	1.15	23 at 0.2 C	60 at 0.2 C	92%@5 C after 2000 cycles	[76]
K_0.03_Cu[Fe(CN)_6_]_0.65_·2.6H_2_O	1.0 M Pb(NO_3_)_2_	1	58 at 0.2 C	68 at 0.2 C	73%@5 C after 2000 cycles	[76]
K_0.03_Cu[Fe(CN)_6_]_0.65_·2.6H_2_O	1.0 M Y(NO_3_)_3_	0.9	60 at 0.2 C	70 at 0.2 C	95%@5 C after 2000 cycles	[76]
Fe[Fe(CN)_6_]_0.73_∙□_0.27_∙3.6H_2_O	0.5 M FeSO_4_	N.A	30 at 40 C	58 at 2 C	80%@10 C after 1000 cycles	[137]

**Fig. 13:**
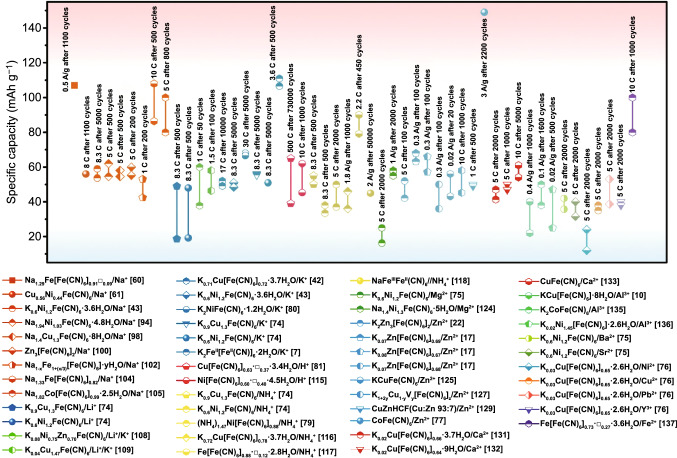
Cycling performance of different PBAs in various aqueous batteries

**Fig. 14:**
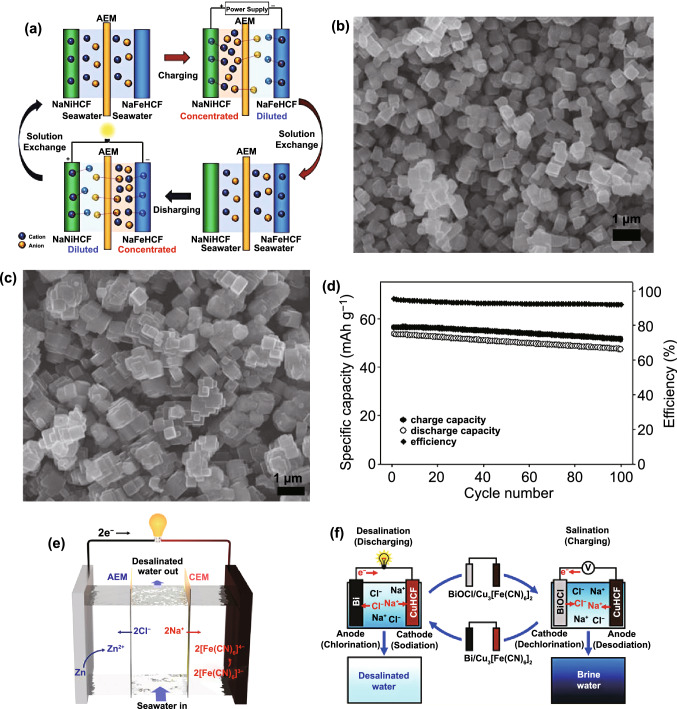
**a** Principle of Na_2_Ni[Fe(CN)_6_]/Na_2_Fe[Fe(CN)_6_] rocking-chair desalination battery. **b** SEM image of Na_2_Ni[Fe(CN)_6_] particles. **c** SEM image of Na_2_Fe[Fe(CN)_6_] particles. **d** Galvanostatic cycling performance of the Na_2_Ni[Fe(CN)_6_]/Na_2_Fe[Fe(CN)_6_] cell in seawater. Reproduced with permission from Ref. [144]. Copyright 2017, American Chemical Society. **e** Schematic of zinc/ferricyanide hybrid desalination flow battery operation during discharge (desalination) and charge (salination). Reproduced with permission from Ref. [145]. Copyright 2018, American Chemical Society. **f** Scheme showing the operation of the Cu_3_[Fe(CN)_6_]_2_·nH_2_O/Bi desalination battery. Reproduced with permission from Ref. [78]. Copyright 2019, American Chemical Society

In addition to the redox potential, the metal atom in high-spin state also has great influence on capacity and cycling stability. In order to more intuitively express the capacity and cyclic stability of different PBAs in aqueous battery, we summarize them in Fig. 13. Obviously, compared with NiFe-PBAs, CuFe-PBAs and ZnFe-PBAs, FeFe-PB, CoFe-PBAs and MnFe-PBAs can provide more capacity because both metals in high- and low-spin state can occur redox reaction. But in terms of cyclic stability, NiFe-PBAs, CuFe-PBAs and ZnFe-PBAs are superior. In addition, defects in the PBAs structure will lead to the reduction of active sites in PBAs, resulting in the decrease of capacity. At the same time, the existence of defects leads to the increase of crystal water in PBAs and thus reduces the cyclic stability. Therefore, Fig. 14 shows that the same-type PBAs have different crystal water content, resulting in different capacities and cycling performance. For the rate performance, high structural stability is beneficial to the rapid insertion and extraction of ions under high current density. Therefore, Table 1 shows that the capacity loss of NiFe-PBAs, CuFe-PBAs and ZnFe-PBAs is less when the current density increases. In addition, the morphology and particle size of PBAs crystals also have a great influence on the rate performance. Generally, PBAs with more uniform morphology and smaller particle size have better rate performance because they have more sufficient contact with the electrolyte and thus are more conducive to rapid ion transport.

Through these relationships between PBAs performance and structure, we can more intuitively select the appropriate PBAs in each aqueous battery. As the capacity provided by NiFe-PBAs, CuFe-PBAs and ZnFe-PBAs in aqueous monovalent metal-ion batteries is limited, after improving the structural stability of FeFe-PB, CoFe-PBAs and MnFe-PBAs, they are more suitable for application in aqueous monovalent metal-ion batteries. For aqueous H^+^ batteries, the high structural stability NiFe-PBAs are a better choice, because the rapid transfer of H^+^ requires high structural stability for host materials. In aqueous NH_4_^+^ batteries, due to the excellent structural compatibility of NH_4_^+^ and FeFe-PB, the application of FeFe-PB has more prominent. For aqueous multivalent-ion batteries, NiFe-PBAs and CuFe-PBAs with higher structural stability are more suitable because of the large hydration ion radius of multivalent ions. Impressively, due to the excellent performance of CoFe-PBAs in aqueous Al^3+^ batteries and Zn^2+^ batteries recently, it is also worth trying in other aqueous multivalent-ion batteries.

## PBAs in Desalination Batteries

To solve the shortage of freshwater source, various physical and electrochemical desalination ways have been studied in the past decades [138–140]. Among physical desalination method, the reverse osmosis, also known as ultrafiltration, is the most representative. The reverse osmosis method is to separate freshwater from seawater by using a semi-permeable membrane which allows only solvent but not solute to permeate. The desalination process can be divided into two steps. The first step is to use a semi-permeable membrane to separate seawater and freshwater on both sides of the membrane, and then, the freshwater moves to the seawater side under the action of osmotic pressure. The second step is to use applied pressure to reverse osmosis the freshwater in the seawater side into the freshwater. Obviously, this physical desalination method has two major disadvantages: the use of high-pressure equipment and regular cleaning and replacement of semi-permeable membrane. In contrast, for electrochemical desalination, such as desalination battery, the desalination is done by the impurity ions hosted in the electrodes, so it does not require an external device. Even in some desalination batteries, semi-permeable membranes may not be used. It is worth noting that the semi-permeable membrane used in desalination battery can be mainly divided into anion exchange membrane (AEM) and cation exchange membrane (CEM). AEM is mainly composed of ion exchange resin and basic active group, and CEM is mainly composed of ion exchange resin and acid active group. Obviously, avoiding the use of semi-permeable membranes will greatly reduce the cost of desalination. Of course, because desalination battery needs to work under the condition of applied voltage, its energy consumption is higher than reverse osmosis. But in terms of simple equipment and environmentally friendly, desalination battery still has great potential. For desalination battery, it consists of cation and anion storage electrodes which remove salt ions from the water through a reversible electrochemical reaction. At present, the electrode materials commonly used in desalination batteries include metal oxide, polyanion polymer, PBAs, etc. Compared with other electrode materials, PBAs mainly have the following advantages as a desalination battery electrode material. Firstly, due to its large ion channel and insertion site, the PBAs can provide higher desalination rate and requires less energy consumption. Secondly, PBAs as desalination battery electrode will greatly reduce the cost due to the simple synthesis method of PBAs. Thirdly, PBAs have better compatibility with multivalent ions in seawater because the crystal water in PBAs structure has a certain shielding effect on the electrostatic repulsion of multivalent ions. In addition to these advantages, PBAs also have a structural disadvantage that needs to be noted. Since most seawater is alkaline, this will reduce the stability of PBAs in seawater and thus affect the desalination performance. However, such structural advantage can be well controlled by controlling PBAs vacancies and crystallinity, so the PBAs-based desalination batteries have received a large number of concerns in recent years.

In desalination battery, the mass transfer resistance of ions in water inevitably leads to the degradation of battery performance, especially when the water concentration is very low. Different from high-concentration electrolytes that are usually defined as concentration higher than 0.1 M, the brackish water (about 500–2000 mg of solute per liter of solution) possesses a small number of accessible ions in the interface of electrode and electrolyte. Therefore, the mass transfer rate of ions in this kind of desalination batteries is slow, and the desalination process can only be guaranteed under the low current density, which is usually about 10 A m^−2^ [141, 142]. In order to introduce the applications of PBAs in desalination batteries more comprehensively, herein the desalination batteries are divided into two groups: the desalination batteries for highly concentrated brine and the desalination batteries for brackish water. The recent advances of PBAs in these two desalination batteries are discussed, respectively, in the following sections.

### PBAs for Highly Concentrated Brine Desalination

For traditional desalination battery like Ag/Na_0.5_MnO_2_ battery, Na^+^ and Cl^−^ are captured in electrode materials through a complex four-step charge and discharge progress and the removal rate of Na^+^ can reach 25% [143]. During the process of discharging (step 1), Na^+^ and Cl^−^ are inserted into the host material by insertion reaction. The deionized water is then exchanged with the water to be treated (step 2). After the above steps, the battery in step 2 is charged so that the ions inserted in discharge reaction can be extracted from the host material (step 3). Then, exchanging the concentrated water with the water to be treated, and the battery continues to perform the discharging process (step 4). Obviously, this desalination process is complex. In order to simplify the desalination process and promote the removal rate of Na^+^, Yoon and co-workers used PBAs to design a simpler and more efficient desalination battery [144]. Figure 14a represents the schematic diagram of PBAs-based desalination battery. The electrode materials used in this desalination battery are highly crystalline Na_2_Ni[Fe(CN)_6_] (NaNiHCF) and Na_2_Fe[Fe(CN)_6_] (NaFeHCF) for better cation capture. The morphology of NaNiHCF and NaFeHCF is revealed in Fig. 14b, c, respectively. In this desalination battery, the water to be treated is divided into two parts by AEM. In fact, the motion of ions during the progress of charging and discharging shows that the desalination battery works in the same way as aqueous rocking-chair battery. Impressively, the use of AEM creates a charge difference between two different solutions of desalination battery, which leads to the concentration and dilution of solutions during charging and discharging without the need to replace the treated water during desalination process, thus simplifying the desalination process. It is noteworthy that NaNiHCF/NaFeHCF full cell demonstrates a good galvanostatic cycling performance with an initial specific capacity about 56 mAh g^−1^ at 0.1 A m^−2^ (Fig. 14d). Meanwhile, this desalination battery possesses an excellent Na^+^ removal efficiency up to 40%. For further improving the desalination performance and promoting its practical application, shortly afterward, Rivest and his co-workers presented a hybrid desalination flow battery that consists of a zinc anode and a PBA cathode [145]. Impressively, this battery possesses a high operation cell voltage of 1.25 V, showing high round-trip efficiency and electrical storage capacity. Figure 14e represents the schematic diagram of hybrid desalination flow battery. Different from above reports of PBAs in desalination batteries, the cathode of this battery is mixed PBAs in liquid form, K_4_Fe(CN)_6_/K_3_Fe(CN)_6_. Na^+^ removal depends on the reaction between K_4_Fe(CN)_6_/K_3_Fe(CN)_6_. Since the liquid-form PBAs possess larger surface, it is more favorable for hosting ions. Thus, impressive salt removal efficiency can be achieved at 85% that is at least two times higher than most desalination batteries reported till now.

As is well known, the membranes play an important role in existing desalination technologies. For reverse osmosis, it requires a semi-permeable membrane for only transporting water molecules but blocking other ions to ensure that the desalination process takes place [146–148]. For conventional desalination batteries, they also need membranes to simplify the desalination steps or improve the performance. Actually, the use of membrane is associated with membrane fouling and replacement cost. Thus, eliminating the need for membrane in highly concentration brine desalination batteries can greatly reduce the cost of desalinations and realize large-scale application of desalination batteries. Since the principle of desalination battery is performed by specific electrode reaction (such as Na^+^ insertion in cathode and Cl^−^ insertion in anode), it possesses the potential to avoid using semi-permeable membrane. Soon after, membrane-free Na_3_Ti_2_(PO_4_)_3_/Bi desalination battery was put forward [149]. Since Cl^−^ removal from BiOCl requires a lower overpotential in acidic conditions than in neutral solutions, the anode reaction is more suitable to perform in acidic conditions. But under acidic conditions, the self-discharge phenomenon of Na_3_Ti_2_(PO_4_)_3_ is serious and it will lead to the spontaneous oxidation of Na_3_Ti_2_(PO_4_)_3_, so it is not conducive to the desalination process of Na_3_Ti_2_(PO_4_)_3_ [150]. Thus, a membrane is still needed to separate the cathode and anode to allow them to work under different acidic conditions. As is well known, CuFe-PBA was demonstrated with excellent cyclability for charge and discharge reactions in acidic media. Therefore, replacing Na_3_Ti_2_(PO_4_)_3_ with CuFe-PBA can achieve membrane-free desalination battery. On the basis of this view, Choi and co-workers constructed a novel desalination battery that uses Cu_3_[Fe(CN)_6_]_2_·nH_2_O as cathode and Bi as anode to enable membrane-free desalination, and Fig. 14f represents the schematic diagram of this rocking-chair desalination battery [78]. This work further promotes the application of PBAs in membrane-free desalination batteries.

### PBAs for Brackish Water Desalination

**Fig. 15:**
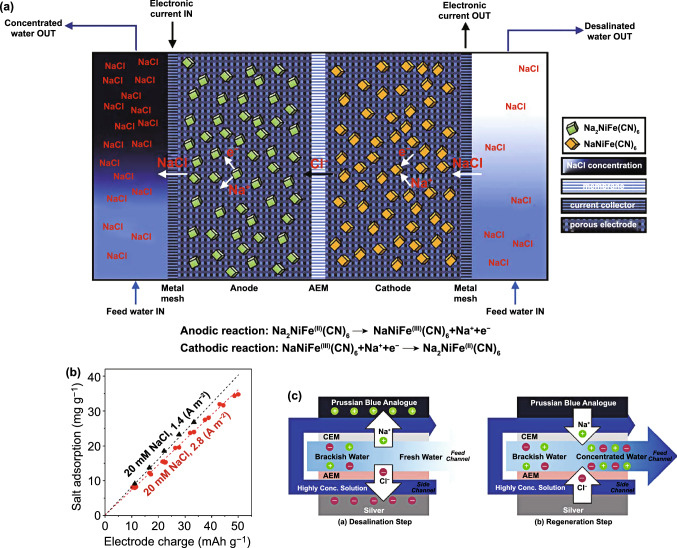
**a** Schematic of NaNiFe(CN)_6_/Na_2_NiFe(CN)_6_ desalination battery operation during discharge (desalination) and charge (salination). **b** Salt adsorption capacity of NaNiFe(CN)_6_. Reproduced with permission from Ref. [158]. Copyright 2017, Elsevier. **c** Schematic of multi-channel NaNiFe(CN)_6_/Ag desalination battery operation during discharge (desalination) and charge (salination). Reproduced with permission from Ref. [161]. Copyright 2019, American Chemical Society

In past studies, researchers have tried various ways to solve the problem of large mass transfer resistance of ions when PBAs-based desalination batteries were used in brackish water. One of the easiest is to control particle size of PBAs [151–157]. Co-precipitation method is a widely accepted route to prepare PBAs. The reaction rate can be slowed down by coordination agents to get less defective PBAs with small size. Based on this view, Smith and co-workers prepared small-sized Na_2_NiFe(CN)_6_ via co-precipitation [158]. Since Na-rich NiFe-PBA does not require a pre-solinization process, they investigated a NaNiFe(CN)_6_/Na_2_NiFe(CN)_6_ battery to treat brackish water as represented in Fig. 15a. Figure 15b shows the salt adsorption capacity of NaNiFe(CN)_6_. For this desalination battery, it only exhibits a 3% sodium removal rate, because the system simply uses granularity control. Although this desalination battery is capable of treating brackish water, its desalination capacity is too poor to meet practical requirements. Later, Logan and co-workers investigated a NaCu[Fe(CN)_6_]/NaCu[Fe(CN)_6_] symmetrical cell to optimize the desalination performance in brackish water [159]. For this desalination battery, it needs to operate at a low current density due to the influence of mass-transfer limitation between electrode and electrolyte. To increase desalination capability, different numbers of ion exchange membranes are added between electrodes to form a hybrid system. Thus, the removal rate of sodium ions is increased to 25%, which demonstrates the feasibility of this system in brackish water. Although these above two desalination batteries are capable of handling brackish water, the fundamental solution is to solve the problem of mass transfer resistance in electrolytes [160]. Then, Yoon and co-workers proposed a multi-channel desalination battery (MC-DB) to solve this problem [161]. Figure 15c exhibits the operation process based on NaNiFe(CN)_6_/Ag desalination battery. Compared with conventional desalination battery with only one feed channel, the MC-DB has two side feed channels and one middle feed channel. The two side feed channels are used for introducing a highly concentrated solution to electrode and one middle feed channel for water desalination. The most innovative part of the battery is that it can adjust the concentration of water around the PBA cathode and Ag anode to reduce the ion migration resistance. Impressively, the MC–DB system shows a desalination capacity of approximately 53 mg g^−1^. Particularly, the maximum salt removal rate can reach up to 0.0576 mg g^−1^ s^−1^ when it treats 10 mM NaCl solution. Therefore, the future application of PBAs-based desalination battery for treating brackish water can be further explored with this battery as a model.

Table 2 summarizes the performance metrics of recently advanced PBAs as desalination battery electrode. As we can see, NaNiHCF/NaFeHCF desalination battery possesses excellent cycle performance with 100 reversible cycles. For desalination capability, the Zn/K_3_[Fe(CN)_6_] cell has the highest specific capacity with 81 and 820 mAh g^−1^ (approximately 86% salt removal) based on the mass of cathode and anode, respectively. Therefore, PBAs-based desalination batteries inherit the potential to be explored. However, only few PBAs can be used in desalination batteries due to poor structural stability of PBAs, such as ZnFe-PBA and MnFe-PBA. Thus, future research can be focused on improving the stability of PBAs to promote more applications of PBAs in desalination batteries. More importantly, we should recognize that this electrochemical desalination technology is a bit too simple for large-scale application. Therefore, in the future exploration of electrochemical seawater desalination, we can develop a hybrid technology that can combine multiple desalination systems to achieve better results.

**Table 2:** Summary of performance metrics of typical desalination batteries

System	Specific capacity (mAh g^−1^)	Cell voltage (V)	Salinity (ppt)	Salt removal (%)	SEC (Wh mol^−1^ NaCl)	Cycles	Refs.
Ag/Na_0.5_MnO_2_	249 (anode) 35 (cathode)	0.30	35	25%	1.9	1	[143]
NaNiFe(CN)_6_/Na_2_NiFe(CN)_6_	59	0	1.2	3%	4.2–16.7	50	[158]
Na_2_Ni[Fe(CN)_6_]/Na_2_Fe[Fe(CN)_6_]	27.5	0.25–0.45	29	40%	1.8	100	[144]
NaCu[Fe(CN)_6_]/NaCu[Fe(CN)_6_]	57	0	2.9	25%	4.1	50	[159]
BiOCl/NaTi_2_(PO_4_)_3_	384 (anode) 63 (cathode)	0.55–0.75	35	20%	31.8 (Na^+^) 95.5 (Cl^−^)	50	[149]
Zn/K_3_[Fe(CN)_6_]	820 (anode) 81.4 (cathode)	1.25	35–100	86%	4.1–8.6	7	[145]

## Conclusions and Outlook

In this review, the recent progress of PBAs as electrode for aqueous monovalent-ion and multivalent-ion batteries is discussed. Meanwhile, the application of PBAs in aqueous desalination batteries is also introduced. PBAs have received a lot of attention for electrode materials in aqueous batteries due to their 3D rigid framework and facile synthesis. Among various host ions, Na^+^ with small hydrated ionic radius and large energy density makes it more suitable for inserting in PBAs lattice. Although Li^+^ is a superstar in non-aqueous batteries, it is unsuitable to be hosted in PBAs in aqueous electrolyte due to its large hydration radius, causing structural damage upon inserting/extracting into/from PBAs. However, co-insertion of Li^+^ with other metal ions possesses great research significance due to synergistic effect. For K^+^, PBAs show excellent rate performance because of the small hydrated ionic radius of K^+^ among monovalent ions. In addition, the application of PBAs in aqueous non-metallic ion battery also exhibits excellent performance. In aqueous H^+^ batteries and NH_4_^+^ batteries, PBAs exhibit excellent rate performance and cycling stability, respectively, due to the special transport mechanism of H^+^ in aqueous electrolyte and the good structural compatibility among NH_4_^+^ and PBAs. As for the application of PBAs in aqueous multivalent ions battery, the vacancies of PBAs can be used as insertion site to contain large volume of hydrated multivalent ions, and crystal water in PBAs also has a certain shielding effect on the electrostatic repulsion of multivalent ions in PBAs structure. Therefore, the application of PBAs in aqueous multivalent-ion battery also has application potential. Although PBAs have a good performance in the application of aqueous monovalent battery, it still has great room for improvement in capacity delivery, cycle stability, rate performance, etc*.* Future research can be considered in the following aspects. (1) The control of vacancies and interstitial water: Although co-precipitation, the most widely used preparation method of PBAs, is simple and efficient in operation, the faster crystallization rate will bring more vacancies and interstitial water. The presence of defects and interstitial water will lead to the reduction of redox sites in PBAs and the stability of PBAs structure, thus resulting in the reduction of capacity and the degradation of cycling performance. Therefore, during the preparation of PBAs, the reaction rate can be reduced by adding chelating agents or adopting multi-step crystallization to improve the crystallinity of PBAs. (2) The suppression of structural change: Generally, PBAs display a transition between one or two crystal types during the insertion and extraction of host ion. Due to the large insertion sites and ion channels of PBAs, such a degree of phase transition will not have a great impact on the structural stability for PBAs. However, for some PBAs such as MnFe-PBAs, the asymmetric distribution of electrons in the d orbitals of Mn^3+^ will lead to the generation of John–Teller distortion, resulting in the phase transformation between three crystal types. This type of structural change has a great impact on the stability of PBAs structure, thus reducing its cyclic stability. In general, this distortion can be well controlled by Mn being partially replaced by Fe to reduce Mn density in PBAs. Therefore, the influence of such multiple-phase reactions on the structural stability of PBAs can be well solved by metal substitution. (3) The enhancement of electronic conductivity: In fact, PBAs as electrode material are poorly conductive. Therefore, it can be combined with various carbon materials or conductive polymers to improve its electronic conductivity. However, these conductive additive agents and binders do not provide capacity in the electrochemical reaction, thus reducing the energy density of PBAs. Actually, improving the conductivity of PBAs on the premise of ensuring the energy density can be solved by directly depositing PBAs onto carbon materials, which are also acted as current collector. This method not only avoids the use of binder and conductive agent, but also achieves the mixing of PBAs and conductive current collector, which has great development potential in improving the conductivity and energy density of the electrode. (iv) The optimization of electrolyte: As shown in Fig. 13, the charging voltage of some PBAs has reached the theoretical decomposition voltage of water. Therefore, for the application of PBAs in aqueous batteries, it is very important to widen the electrochemical stability window of electrolyte. At present, the simplest and most effective method is to increase the solute content in the electrolyte and enhance the interaction between anions/cations and water molecules to reduce the content of free water molecules, so as to widen the electrochemical stability window of electrolyte. In addition, as shown in Eq. 3, under alkaline conditions, the dissolution of PBAs will be accelerated, especially the dissolution of PBAs containing Co, V, etc*.*, which will produce toxic components in the electrolyte. Therefore, acidic electrolyte should be selected in general to improve the stability of PBAs and avoid the generation of toxic components.

For the application of PBAs in aqueous multivalent-ion battery, besides the four points discussed above, there is another aspect that needs special attention. Although the crystal water in PBAs has a certain shielding effect on the electrostatic interaction between multivalent ions and PBAs, the presence of crystal water has negative impact on the structural stability and capacity delivery of PBAs. Therefore, it becomes important to explore the balance between the content of crystal water and the cycling life of PBAs. In addition, most of the current researches on the reaction mechanism of aqueous battery are based on ex situ characterization methods. Therefore, it is also important to use a variety of in situ characterization methods to explore the insertion/extraction mechanism of host ions.

As desalination battery is a special kind of aqueous battery, the development of PBAs in conventional aqueous battery also promotes its application in desalination battery. Among various PBAs, Zn/K_3_[Fe(CN)_6_] shows the best performance, which widens the way for the future research of PBAs in desalination battery. Moreover, PBAs-based multi-channel desalination battery also shows excellent salt removal efficiency in brackish water through special structural design, which provides a clear direction for desalination battery in brackish water in future. In general, PBAs as a desalination battery electrode have the following advantages: (i) For PBAs, they have large ion channels and well electrochemical reversibility, which enable PBAs-based desalination battery to achieve better desalination performance with simplified desalination steps. (ii) PBAs have good chemical stability under acidic conditions, which enables it to form a practical desalination battery with traditional Cl^−^ storage electrode and realize the membrane-free. (iii) Except for large ion channel, PBAs also have small particle size, which makes it suitable to purify brackish water in desalination battery. For the future application of PBAs in desalination batteries, the simplification of desalination steps and the development of membrane-free desalination batteries are the promising directions. Furthermore, in order to get better desalination performance, it is suggested to take the following methods: (i) preparing high-crystallinity, low-defect and small-sized PBAs, (ii) exploring PBAs with new structure, such as MnMn-PBA, CoCo-PBA and CrCr-PBA and their derivatives, and (iii) combining PBAs-based desalination battery with other systems to form hybrid desalination system.
