# Correction to: Prussian Blue Analogues in Aqueous Batteries and Desalination Batteries

**DOI:** 10.1007/s40820-021-00715-2

**Published:** 2021-09-04

**Authors:** Chiwei Xu, Zhengwei Yang, Xikun Zhang, Maoting Xia, Huihui Yan, Jing Li, Haoxiang Yu, Liyuan Zhang, Jie Shu

**Affiliations:** School of Materials Science and Chemical Engineering, Ningbo University, Ningbo, Zhejiang 315211 People’s Republic of China

## Correction to: Nano-Micro Lett. (2021) 13: 166 10.1007/s40820-021-00700-9

In the published article the authors equal contribution statement is incorrect in the foot note. The correct statement should be read as “Chiwei Xu and Zhengwei Yang have contributed equally to this work”.

